# SomatiCA: Identifying, Characterizing and Quantifying Somatic Copy Number Aberrations from Cancer Genome Sequencing Data

**DOI:** 10.1371/journal.pone.0078143

**Published:** 2013-11-12

**Authors:** Mengjie Chen, Murat Gunel, Hongyu Zhao

**Affiliations:** 1 Program of Computational Biology and Bioinformatics, Yale University, New Haven, Connecticut, United States of America; 2 Department of Genetics, Yale University, New Haven, Connecticut, United States of America; 3 Department of Neurosurgery, Yale University, New Haven, Connecticut, United States of America; 4 Department of Biostatistics, Yale University, New Haven, Connecticut, United States of America; Deutsches Krebsforschungszentrum, Germany

## Abstract

Whole genome sequencing of matched tumor-normal sample pairs is becoming routine in cancer research. However, analysis of somatic copy-number changes from sequencing data is still challenging because of insufficient sequencing coverage, unknown tumor sample purity and subclonal heterogeneity. Here we describe a computational framework, named SomatiCA, which explicitly accounts for tumor purity and subclonality in the analysis of somatic copy-number profiles. Taking read depths (RD) and lesser allele frequencies (LAF) as input, SomatiCA will output 1) admixture rate for each tumor sample, 2) somatic allelic copy-number for each genomic segment, 3) fraction of tumor cells with subclonal change in each somatic copy number aberration (SCNA), and 4) a list of substantial genomic aberration events including gain, loss and LOH. SomatiCA is available as a Bioconductor R package at http://www.bioconductor.org/packages/2.13/bioc/html/SomatiCA.html.

## Introduction

During carcinogenesis, there are often alterations of the dosage and/or structure of tumor suppressor genes or oncogenes in cancer cells through somatic chromosomal alterations. Identifying genomic regions with recurrent copy number alterations (gains and losses) in tumor genomes is an efficient way to find cancer driver genes [1]. Ideally, such characterization should include both the precise identification of the chromosomal breakpoints of each alteration and the absolute estimation of copy numbers in each chromosomal segment. Earlier studies used oligonucleotide microarrays to infer genome-wide copy-number changes. Recent advances in massively parallel sequencing provide a powerful alternative to DNA microarrays for detecting copy-number alterations [2]. The advantages of sequencing-based approaches include its comprehensive and unbiased survey of all genomic variations [3] and ability to detect both copy number aberrations (CNAs) and single nucleotide variations (SNVs) simultaneously in each sample, which offers critical information for our understanding of cancer genome evolution.

Many algorithms have been developed to detect copy number variations (CNVs) from whole genome or exome sequencing data, such as methods using raw read-depth [2]–[5], read-pair alignment [6], [7], split-read mapping [8], [9] and assembly-based (AS) methods [10], [11]. However, these methods are not well suited to infer absolute somatic copy-number because they are developed to analyze data from normal instead of tumor samples. Compared to normal samples, tumor samples have some unique features including: (i) an unknown fraction of normal cells (admixture rate) that are nearly always intermixed with cancer cells; and (ii) the heterogeneity of cancer cell population owing to ongoing subclonal evolution. Although some methods have been developed for Somatic CNA (SCNA) identification in whole cancer genome sequencing, most of them do not explicitly model tumor purity [12], [13]. For those accounting for tumor purity, ExomeCNV [14] estimates the admixture rate based on the largest Loss of Heterozygosity (LOH) region in a genome, which likely produces a biased estimation. A more commonly used option in ExomeCNV is a default setting of 0.3 for the admixture rate. Control-FREEC [15] requires a prior specification of the normal contamination level or a pre-specified ploidy to estimate the normal contamination through the median shift of copy number in altered regions towards the normal baseline. Both methods have low tolerance to contamination. Algorithms developed on arrayCGH data, such as ASCAT [16] and ABSOLUTE [17], are specialized to estimate tumor purity but do not provide a comprehensive framework for subclonality identification or segment calling.

**Figure 1:**
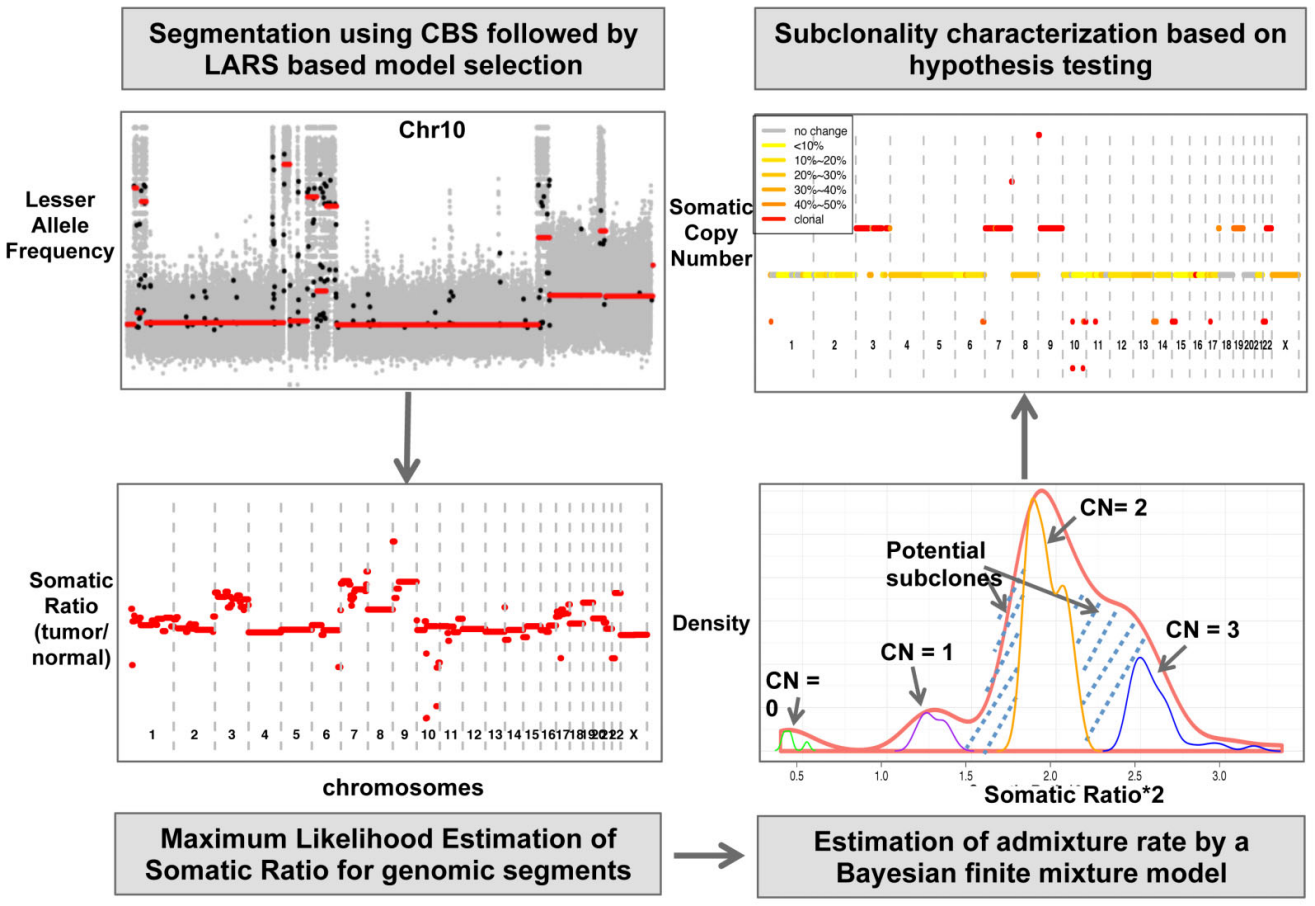
Overview of SomatiCA framework. First, SomatiCA segments the genome and identifies candidate CNAs utilizing both read depths (RD) and lesser allele frequencies (LAF) from mapped reads. Second, SomatiCA estimates the admixture rate from the relative copy-number ratios of a tumor-normal pair by a Bayesian finite mixture model, which has high tolerance on contamination from normal cells. Finally, SomatiCA quantifies somatic copy-number and subclonality for each genomic segment to guide its characterization.

Here we present SomatiCA, a novel framework that is capable of identifying, characterizing and quantifying SCNAs from cancer genome sequencing (Figure 1). By directly accounting for tumor purity and subclonality, SomatiCA was specially developed to analyze tumor samples with contamination and/or heterogeneity. First, SomatiCA segments the genome and identifies candidate CNAs utilizing both read depths (RD) and lesser allele frequencies (LAF) from mapped reads. Second, SomatiCA estimates the admixture rate from the relative copy-number ratios of a tumor-normal pair by a Bayesian finite mixture model, which has high tolerance on contamination from normal cells. Finally, SomatiCA quantifies somatic copy-number and subclonality for each genomic segment to guide its characterization. Results from SomatiCA can be further integrated with SNVs from the same sequencing experiment to gain a better understanding of tumor evolution.

## Results

### Segmentation strategy in SomatiCA

Although next generation sequencing (NGS) technology generates data with higher resolution than SNP arrays and array comparative genomic hybridization (aCGH), the signal is complicated by mappability, GC-content, alignment bias and other issues [15]. This makes the analysis of NGS data not just a direct adaptation of existing methodologies on aCGH but an extension requiring extra care on many factors affecting data analysis and interpretation. For example, after quality control and de-noising, many existing NGS CNV calling tools directly apply methods developed for aCGH data [14]. However when we applied CBS [18], a commonly used method for aCGH data, we found it was very sensitive to fluctuation in NGS signals and reported change points likely to be false positive (see simulation results).

In contrast, SomatiCA implements a smoothing-based de-noising step to reduce the effects of outliers from input LAF (Figure S1). Given the initial change points detected by CBS, we implemented a variable selection procedure to remove change points that are likely to be false positives. This is accomplished in SomatiCA by using CBS detected change points as the predictors for the input LAF and then performing variable selection via Bayesian Information Criterion (BIC) based on a LARS [19] solution path. For the selected change points, SomatiCA further assesses whether they capture the changes in somatic copy-numbers. To quantify these changes, we define somatic ratio as the RD ratio of the tumor to the paired normal in a segment (with identical coverage in the tumor and normal sample assumed). SomatiCA derives a Maximum Likelihood Estimate (MLE) of the somatic ratio for each segment using RD information from all paired SNPs in that segment. Two adjacent segments are merged if the difference in the somatic ratios is less than T, which is a tuning parameter in the implementation with a default value of 0.05, equivalent to 5% change in somatic copy-number without normal contamination. The MLEs of the somatic ratio for the refined segments are recalculated. This refinement procedure is applied repeatedly until no adjacent segments have somatic ratio difference less than T. In SomatiCA, information from both germline heterozygous and homozygous SNPs are utilized. LAF on heterozygous sites are used in the initial segmentation. RD on heterozygous and homozygous sites are used to calculate the somatic ratios.

### Simulation Strategy

We perform simulations to evaluate the statistical power of SomatiCA and for comparisons with other methods. In the absence of validated biological datasets, such simulation studies may yield insights on the pros and cons of different methods. However, because of the complexity of the genome and the sequencing process, e.g., the non-uniform distribution of RD across the genome in NGS, it is non-trivial to simulate cancer sequencing data that capture the complexity in real NGS data. Inspired by Ivakhno et al [12], we utilized a normal sample (denote as GLI-N1, unpublished data) to simulate the cancer sequencing data as follows (scripts in Text S1):

1. Duplicate the RD and lesser allele counts from the GLI-N1 sample.
2. For each 10 kb genomic window, estimate the median and standard deviation of RD of all sites and lesser allele counts of all heterozygous sites.
3. At predetermined positions, place SCNA events ranging from 10 kb to a whole chromosome, with varying magnitudes of changes including double deletions, LOH, 1 and 2 copy number gains (as well as different subclonalities including 20% and 40%). Each aberration contains at least 5 heterozygous sites.
4. Simulate SCNA events by altering the medians in corresponded windows.
5. Simulate RD and lesser allele counts in SCNA events windows through normal distributions with means equal to the altered medians resulted from step 4) and standard deviation equal to the estimates from step 2).
6. Admix pseudo cancer counts and normal counts with a gradient of the admixture rate, 0.2, 0.4 and 0.6.
7. In addition to the actual RD reported in GLI-N1 (∼60×), simulate read depths of 40× and 20× by randomly removing a proportion of reads.

In total, we simulated 90 cancer genomes (3 admixture rates* 3 coverage*10) and each of them contained 40 SCNAs.

### SomatiCA effectively reduces false positive rate in the segmentation

We applied SomatiCA to these simulated data to evaluate the performance for SCNA detection under different scenarios. We compared its performance with CBS and cumSeg [20], a similar segmentation method using model selection to identify change points with a different initial over-detection step. For fair comparisons, we applied the same smoothing and refinement procedure as implemented in SomatiCA for both CBS and cumSeg. Considering that CBS and cumSeg do not adjust for admixture rate, we used a lenient criterion to determine whether a SCNA call was a positive discovery. If the somatic ratio was less than 0.8 or greater than 1.2, the corresponding segment was reported as an genomic region with somatic gain or loss. For a true positive SCNA call, we required the detected breakpoints within 100 kb of true ones.

**Figure 2:**
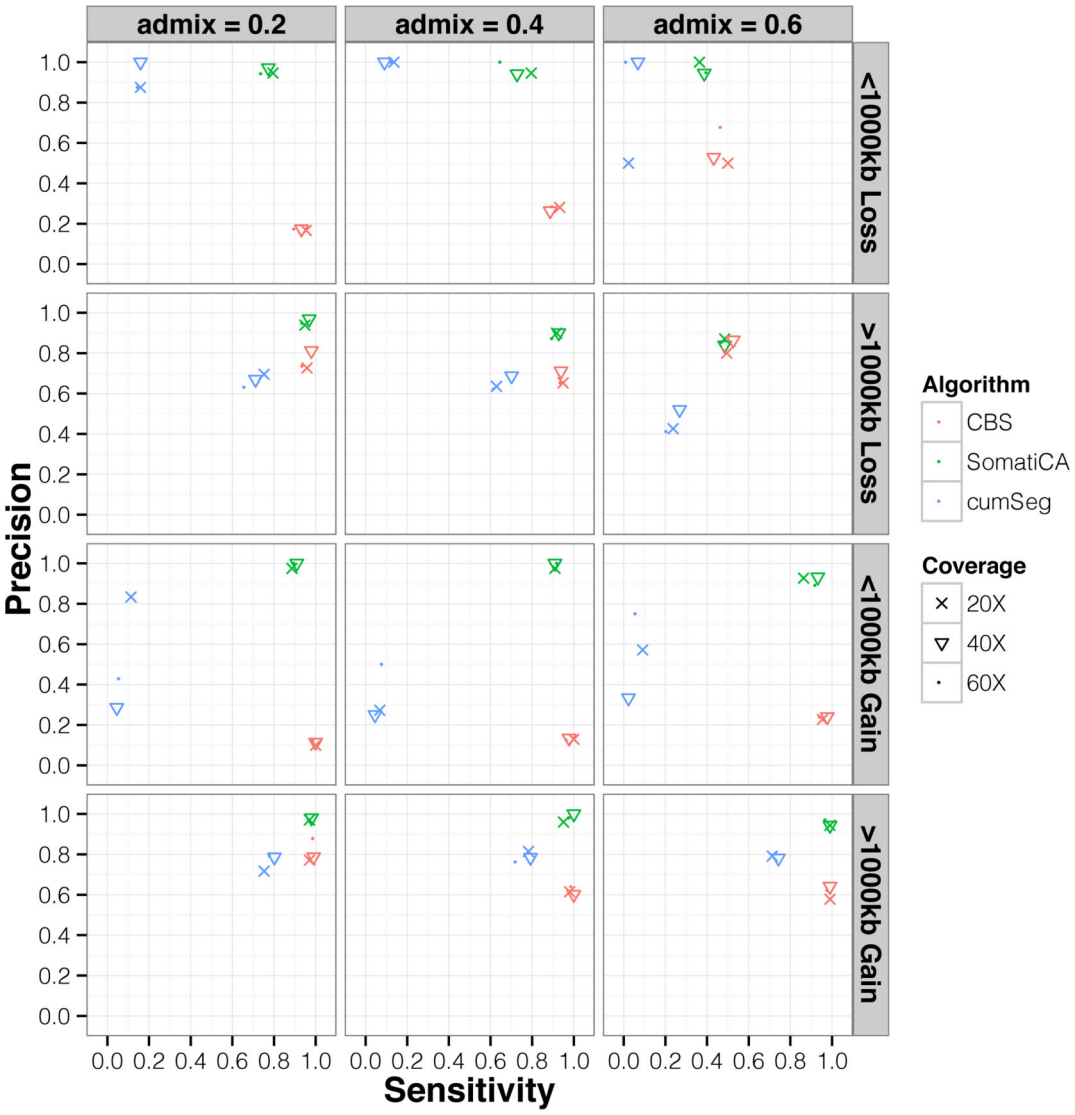
Precision Vs. Sensitivity comparison of three segmentation methods. Summary of precision and sensitivity over 90 simulated cancer genomes with different admixture rates and coverage. CBS and SomatiCA outperformed cumSeg in sensitivity at detecting SCNAs larger than 1% false positive calls whereas SomatiCA achieved higher precision. For SCNAs smaller than 1 Mb, CBS still maintained a high sensitivity of 98% but over 60% of CBS calls were false positives. Both SomatiCA and cumSeg used model selection to effectively reduce the false positive rate with some compromise on sensitivity.

Overall, CBS and SomatiCA outperformed cumSeg in sensitivity at detecting SCNAs larger than 1 Mb (Figure 2). However, CBS had 30% false positive calls whereas SomatiCA achieved higher precision. Moreover, CBS tended to over-detect breakpoints on the same alteration. On average CBS reported 1.82 segments for a ∼1 Mb event and 3.15 segments for a ∼10 Mb events. In contrast, SomatiCA and cumSeg reported 1.01 and 1.07 segments for the SCNAs larger than 1 Mb. This improvement is due to the model selection step for change points that removes those showing small fluctuations, which more likely result from the same aberration.

For SCNAs smaller than 1 Mb, CBS still maintained a high sensitivity of 98% but over 60% of CBS calls were false positives. Both SomatiCA and cumSeg used model selection to effectively reduce the false positive rate with some compromise on sensitivity. SomatiCA detected 83% simulated SCNAs whereas cumSeg only captured 10%. We note that penalization through model selection is only one of many reasons for the lower sensitivity in smaller SCNAs identification. Because SomatiCA segments the genome only based on LAF from heterozygous sites, it may overlook the aberrations with fewer heterozygous sites. On chromosomes 3 to 15 in the GLI-N1 sample, which we used as the template for simulation, the distances between adjacent heterozygous sites ranged from 5 bp (1% quantile) to 17,036 bp (99% quantile) with a median of 453 bp. The number of heterozygous sites within the undetected SCNAs ranged from 6 to 76 with a median of 22. Strong dependency on the number of heterozygous sites is a major drawback of all approaches using LAF (or BAF) in chromosome segmentation. The nonuniform coverage and errors signal in sequencing data makes it challenging to make inference with only a few markers. In practice, we suggest to use RD based methods as complementary approaches to cover a wider range of SCNA events (as elaborated more in the discussion).

When the contamination from normal cells increased over 50% (admixture rate = 0.6), all three methods suffered in power and precision on detecting copy loss or gain. For example, when the admixture rate is 0.6, the expected somatic ratio for one copy loss and one copy gain is 0.8 and 1.2. Thus the cutoff values used in the previous comparisons may be too stringent to identify SCNA events. This suggests the importance of adjusting parameters for the admixture rate in SCNA calling.

### Explicit modeling of admixture rate

As we mentioned, an unknown fraction of normal cells and the heterogeneity of cancer cell population are two factors requiring special attention in the analyses of tumor samples. We begin by explaining how the admixture rate would affect SCNAs calling using a hypothetical example. For a tumor sample with 0, 1, 3 and 4 copies at different chromosomal segments is intermixed with 40% of a paired normal sample with 2 copies, the expected somatic ratios are 0.4, 0.7, 1.3, and 1.6, respectively. Without any adjustment for the admixture rate, the inferred copy-numbers would be 1, 2 (or 1), 2 (or 3), and 3, respectively. In this case, double deletions would be mistakenly called as LOHs, whereas true LOHs would be nearly undetectable resulting in inaccurate inference on copy numbers. One key observation here is that there is an overall shift of the expected somatic ratios from the ones without any contamination, and this general shift could be utilized to infer the admixture rate. However, there are two complications to capitalize on this observation: first, the types of SCNAs are unknown (e.g. there are 4 types in our hypothetical example); second, the presence of subclonal SCNAs may further complicate the somatic ratio profile and consequently affect the copy number. To address these issues in a coherent manner, we have developed a probabilistic model under a full Bayesian framework as detailed below.

The basic idea behind admixture rate estimation in SomatiCA is that the somatic ratios of clonal segments are centered around a certain discrete level whereas those of subclonal segments have no constraints. Therefore based on its somatic ratio, each genomic segment can be either assigned an integer copy-number or classified as a subclonal event. The proportion of intermixed normal cells can be estimated from the shift of somatic ratios of clonal SCNAs from their expectations in the pure and homogeneous tumor samples. To accomplish this, we first estimated the most likely number of components from the input somatic ratio distribution, then fitted a Bayesian finite mixture model to assign copy number to each segment based on the corresponding posterior probability, and finally we estimated the admixture rate by an optimal solution contributed by explanation of the copy number shift of all clonal segments from integer levels.

Our model is similar to ABSOLUTE [17], a Gaussian mixture model to identify tumor purity and ploidy on arrayCGH or low-pass sequencing data, with the major differences on assumptions being: 1) ABSOLUTE assumes a uniform distribution on subclonal events; in SomatiCA, subclonal events are identified based on the posterior probabilities, i.e., the departure from integer copy numbers; 2) ABSOLUTE constrains the genomic mass allocated to each copy-state while SomatiCA not. Moreover, these two methods take different quantities as input. ABSOLUTE takes the copy-ratio as input, a quantity measures the local DNA dosage conditioning on the aneuploidy of the tumor, whereas SomatiCA uses the somatic ratio, which is an absolute measure between normal and tumor samples without conditioning on the global measure of tumor ploidy (identical coverage for two libraries is assumed). The usage of the somatic ratio frees SomatiCA from the estimation of ploidy. Instead of searching all feasible combinations of ploidy and admixture rate, SomatiCA only searches for a solution of admixture rate with the somatic ratio of 1 corresponding to the integer copy number of 2.

**Figure 3:**
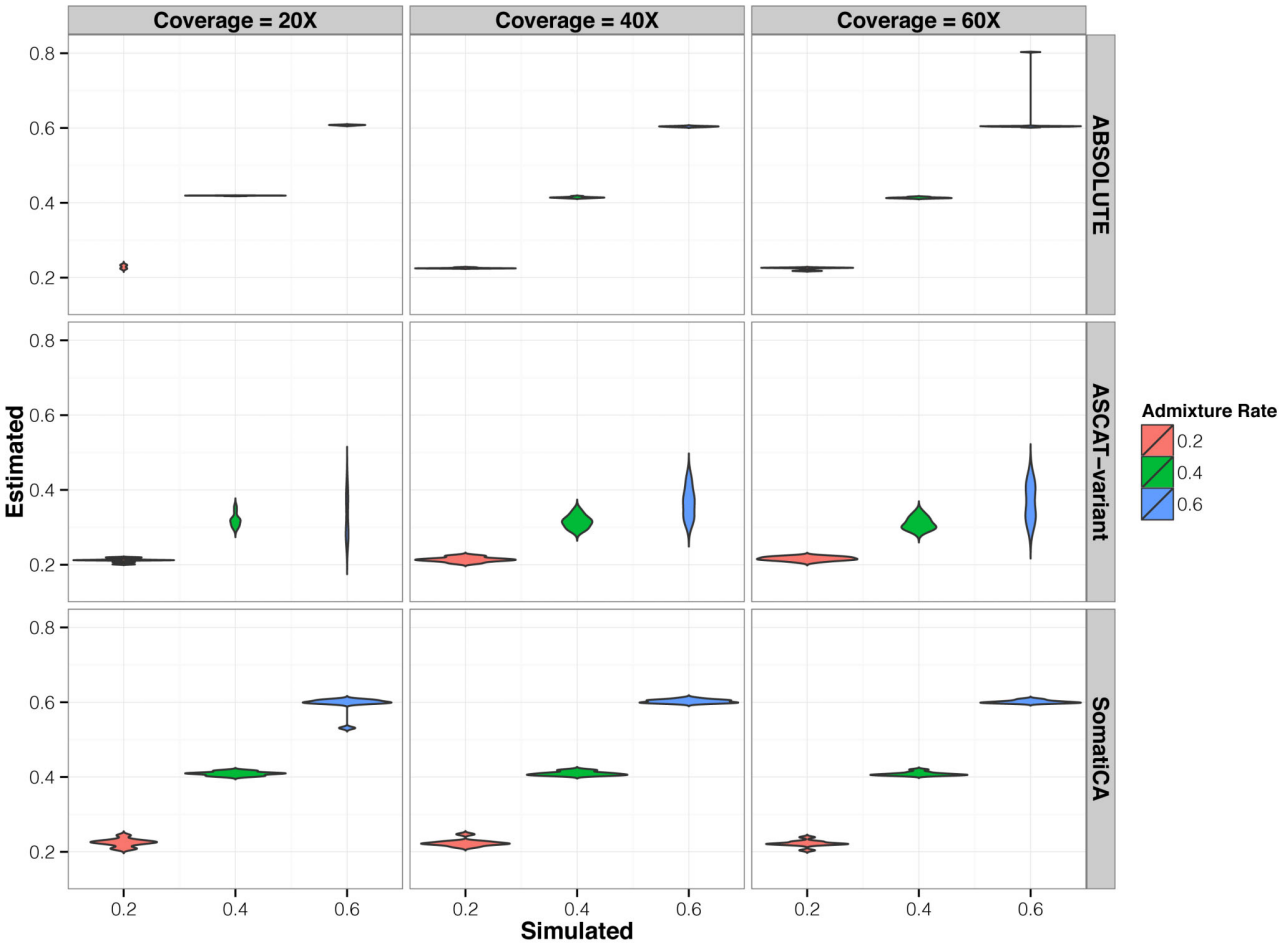
Boxplot of the admixture rate estimation using SomatiCA, ABSOLUTE and ASCAT-variant. Both SomatiCA and ABSOLUTE outperforms ASCAT-variant. SomatiCA achieves comparable performance as ABSOLUTE with few constraints and less computational burden.

We evaluated the performance of our method using 90 simulated cancer genomes. SomatiCA generated accurate estimation of the admixture rate even when the coverage was as low as 20×. As a comparison, we also estimated the admixture rate by ABSOLUTE and a variant of ASCAT. ASCAT uses BAF and logR ratio (conditioning on the aneuploidy of the tumor) to estimate tumor ploidy and purity, which is not directly applicable to our data. In our comparisons, we used a variant of ASCAT algorithm that maintained its main features: we calculated the total distance to an allelic integer copy number solution for each segment and summed over all segments; then we searched for a solution of the admixture rate that minimized the total distance. For ABSOLUTE, among top five possible combinations of admixture rate and ploidy (by likelihood), we selected the one with the copy ratio of 1 corresponding to the integer copy number of 2 as the final solution. The results summarized in Figure 3 show that SomatiCA has a comparable performance with ABSOLUTE and outperforms ASCAT.

We think two reasons contributed to the better performance of SomatiCA compared to ASCAT-variant. First, ASCAT estimates the integer copy number for each segment using the integer closest to the observed somatic allelic copy. When the admixture rate is high, this approximation is problematic. For example, when the admixture rate is 0.6, the somatic copy of double deletion is 1.2. The integer copy number for this double deletion event is assigned as 1 instead of 0. In contrast, SomatiCA pre-calculates the number of possible discrete levels from the histogram of the somatic ratios and assigns the integer copy number based on the order of its discrete level using the level of 2 copy as a reference. Hence, it is still capable of estimating the absolute copy number well with high accuracy when the admixture rate is high. Second, ASCAT optimizes over all the SNPs, whereas SomatiCA takes into account the influence of intra-tumor subclonal heterogeneity and only optimizes over clonal events. This approach compensates for the underestimation from the optimization with all segments.

Moreover, SomatiCA achieves comparable performance as ABSOLUTE with few constraints and less computational burden. SomatiCA does not constrain the genomic mass allocated to each copy-state, or the relative proportion of subclones. Potential subclones, identified by low posterior probabilities, are excluded from admixture rate estimation. With the assumption of copy ratio of 1 corresponding to the integer copy number of 2, SomatiCA only optimizes over one parameter — admixture rate, which reduces the burden of simultaneous estimation of admixture rate and ploidy. The average CPU running time for the admixture rate estimation in SomatiCA is 27.5 seconds (5000 MCMC steps) whereas that for ABSOLUTE (ploidy ranged from 0.95 to 4) is 450 seconds. In SomatiCA, the ploidy could be estimated by averaging copy-number over the genome after adjusting for the admixture rate.

We further looked into the simulated genomes with high normal contaminations where the admixture rate was 0.6. We inferred the copy number for SCNAs detected from these simulated genomes with adjustment using estimated admixture rate from SomatiCA, and compared the results with the copy number inferred without any adjustment, and those with adjustment using an admixture rate of 0.2 and those using 0.4. As shown in Figure S2, the estimation from SomatiCA helped to increase the accuracy of the inferred copy number inference for SCNAs compared to setting admixture rate at pre-specified (and incorrect) levels.

### Subclonality characterization

The presence of genetic diversity within tumor samples, that is, subclonality, offers important clues to tumor evolution. Accurate inference of copy number status through adjustment of admixture rate provides opportunities for SomatiCA to identify subclonal alterations against the background of the predominant ones. SomatiCA characterizes the subclonality for each segment through performing hypothesis testing. It first calculates the copy number for each segment in the control normal sample 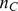. Then it tests whether copy number change in the corresponding tumor sample can result in a change of exactly one copy of one allele. In our simulation study, we placed 4∼5 SCNAs (larger than 10 Mb, subclonal percentage of 0.2 or 0.4) on chromosome 12 to 15 in each simulated cancer genome. In total, for each combination of admixture rate and coverage, there are 46 true positive subclonal events across ten simulated cancer genomes. The subclonal calls from other chromosomes are false positives, resulting from either an underestimation of clonal events or a misclassification of copy number neutral event. When the admixture rate is 0.2 or 0.4, SomatiCA recovered 87% of true subclonal events (40 out of 46) and reported 8 false positives on average. When the admixture rate is 0.6, SomatiCA was still able to recover 84% of true subclonal events but reported 20 false positives. 95% of false positives subclonal events are misclassified from copy number neutral events. This result indicates that SomatiCA achieves high precision on detecting clonal events. However when the admixture rate gets higher, more false positive calls would emerge from misclassification of copy number neutral events.

### Application to TCGA benchmark 4 data

We used the TCGA mutation calling benchmark 4 datasets to evaluate the performance of SomatiCA and others on real data. This whole genome sequencing benchmark dataset is ideal for such an evaluation because it consists of artificially mixed samples with the proportion of tumor samples in a gradient from 20% to 95%. We focused our analysis on 7 mixed HCC1143 samples sequenced at 30× (Table 1). For each mixed sample, we first performed segmentation implemented in SomatiCA and calculated the somatic ratios using HCC1143 30× normal sample as a matched pair. We adjust the median of tumor library so that the medians of two were the same. Then we input somatic ratios to SomatiCA, ASCAT-variant and ABSOLUTE. For each sample, ABSOLUTE output 19 feasible combinations of admixture rate and ploidy (the allowed range of ploidy set to be 0.95 to 4) that covered a broad range. Take sample HCC1143.n60t40 as an example (60% normal cells mixed with 40% tumor cells), the estimated admixture rate is ranged from 0.32 to 0.84. To match the underlying assumption in SomatiCA, we manually selected ABSOLUTE solutions with the copy ratio of 1 corresponding to the integer copy number of 2 (or 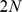). However we note that selected ABSOLUTE solutions under such criteria are more precise than solutions with top SCNA-fit log-likelihood score. We summarize the described estimations in Table 1. Overall, SomatiCA has a comparable performance to ABSOLUTE. Both outperform ASCAT-variant. In three replicate samples with 25% contamination from normal cells (though different spike-in SNVs introduced), SomatiCA produced more precise and stable estimations. This result suggests that the correspondence of 1 to integer copy number of 2 may be a fair assumption to make in cancer sequencing data with a paired normal sample sequenced at a comparable depth.

**Table 1:** Admixture Rate Estimation for the TCGA benchmark data.

Normal Mixing Fraction	Subclone*	SomatiCA	ABSOLUTE	ASCAT-variant
0.05	0	0.18 (0.020)	0.22	0.09
0.2	0	0.24 (0.015)	0.28	0.12
0.4	0	0.34 (0.026)	0.39∼0.46	0.17
0.6	0	0.54 (0.017)	0.52∼0.62	0.30
0.25	0.05	0.23 (0.019)	0.43	0.14
0.25	0.1	0.26 (0.028)	0.17∼0.36	0.12
0.25	0.4	0.23 (0.021)	0.37	0.17

This table shows the estimated admixture rate for a series of artificial mixed HCC1143 samples from SomatiCA, ABSOLUTE and ASCAT-variant. For SomatiCA, the estimate is the mean from five independent MCMC runs with standard deviation shown in parenthesis. For ABSOLUTE, the solution with the copy ratio of 1 corresponding to or around the integer copy number of 2 (or 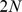) is shown. If the solution is not unique, a range for possible solutions is shown.

* Subclones are only introduced as SNV (or SV) not CNA.

**Figure 4:**
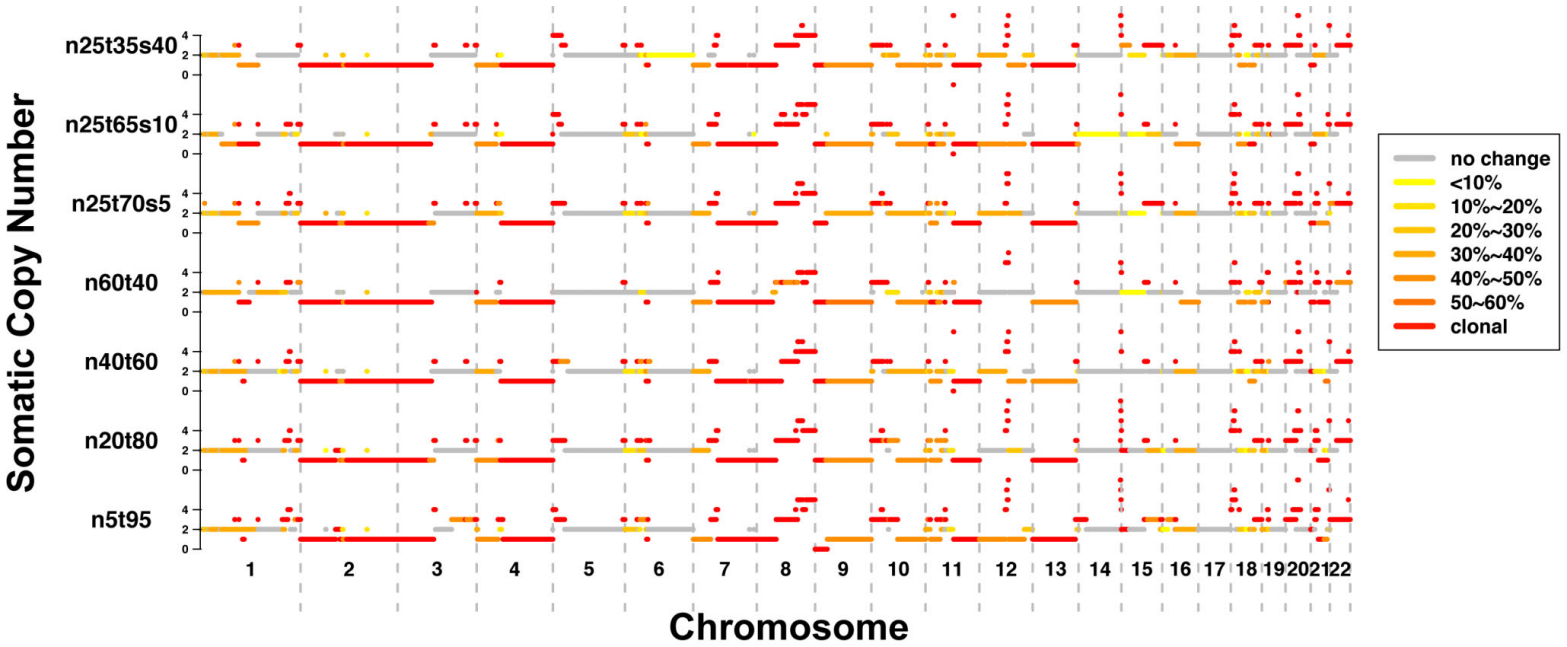
Somatic copy number and subclonality characterization for TCGA benchmark HCC1143 samples. The calling result is consistent across samples with different mixing proportion of normal cells, which demonstrates the robustness of SomatiCA to different extent of contamination.

After adjusting for estimated admixture rate, we used SomatiCA to call SCNAs for these samples. Figure 4 shows the somatic copy number and subclonality characterized for 7 samples we analyzed. The result is consistent across samples with different mixing proportion of normal cells, which demonstrates the robustness of SomatiCA to different extent of contamination. However, due to the potential model overfitting and unavoidable identifiability issue, SomatiCA does not report any admixture rate over 80%. For TCGA benchmark 4 sample HCC1143.n80t20 and HCC1143.n95t5 (mixed with 80% and 95% normal cells), SomatiCA only reported segmentation results without adjusting for admixture rate.

### Application of SomatiCA to a GBM sample

We applied SomatiCA to the whole genome sequencing data on the Complete Genomics platform of a patient with diagnosed primary glioblastoma (GBM) (unpublished data). In Figure S3 and S4, we show the segmentation from SomatiCA and its comparison with CBS and cumSeg using chromosomes 7 and 10 respectively. The estimated admixture rate for this sample was 37.1%. After adjusting for the admixture rate, we identified 121 SCNAs with sizes ranging from 3428 bp to a whole chromosome. These SCNAs included one copy gain on whole chromosome 7, one copy gain for whole chromosome 9, and both LOHs and copy-neutral LOHs on chromosome 10. We further compared these SCNAs with 20 known GBM drivers listed in [21] and found that these SCNAs showed overlap with 15 out of 20 known GBM drivers. Among these, the amplification on CDK6, EGFR and MET, and the deletion on NF1 are clonal whereas other events are subclonal.

## Discussion

In this article, we have described a novel computational framework, SomatiCA, to identify SCNAs from cancer sequencing data. It was developed to address contamination and heterogeneity in tumor samples, two major challenges in cancer genome analysis. Extensive simulations have demonstrated the better performance of our methods over the existing ones.

SomatiCA has been implemented as four functional modules in R: initial segmentation, estimation of somatic ratio with segmentation refinement, adjusting for admixture rate and subclonality characterization. Each module in SomatiCA can be called independently. It is straightforward to implement customized procedure incorporating one or all modules from SomatiCA. Although the data motivating the development of SomatiCA were generated from the Complete Genomic platform, the input to SomatiCA is the RD and LAF for all the paired SNP sites, making it generally applicable to analyze the data from other platforms. SomatiCA is also scalable because the segmentation on different chromosomes can be paralleled (See Text S2 for a manual of SomtiCA package).

Despite many advantages, we do note that there are several caveats for using SomatiCA.

First of all, SomatiCA requires mapping to a reference genome and genotype calling as pre-processing steps. It has been shown that mappability, GC-content bias and quality control measure of reads all affect read depths thus CNV calling [22]. Although the impacts of these issues may be reduced in SCNA calling with paired normal-tumor samples to some extent, special cautions are still needed regarding to the choice of aligners, mapping quality filters and genotype callers. Sequencing depth may also affect the performance of SomatiCA. SomatiCA was developed on the sequencing data with a decent coverage of 30× or higher. For low coverage samples (for example, 0.01–0.5×), we recommend specialized methods such as BIC-seq [23] and CNAnorm [24].

Secondly, the segmentation in SomatiCA relies upon the change points detected by CBS. In a recent study, Cai et al [25] reported that CBS had deficiency in the detection of sparse and short segments with interval lengths less than 40 data points. It has also been shown in our simulation studies that segments with only a few markers tend to be overlooked by CBS and thus by SomatiCA. Low sensitivity on short segments is further exacerbated by the usage of the diluted signal from heterozygous sites. Therefore, SomatiCA, as currently implemented, may not be suitable for sparse and short segment discovery in cancer sequencing data. This is a common issue for the methods using BAF (LAF). According to a survey of 3131 cancer samples, the median length of focal SCNAs was reported to be 1.8 Mb (range of 0.5 kb–85 Mb). To identify a wide range of SCNAs from several hundred base pairs to even a chromosome, we recommend to consider complementary approaches in practice. The segmentation method in SomatiCA falls into the category of global approaches, which call break points through testing against the background of an entire chromosome. Local approaches, which refer to those methods that aim to identify SCNAs by comparing the RD in the tumor genome with that of the matched normal genome at each genomic position (or window), such as BIC-seq [23], CNVseg [12] or SegSeq [2], may help to identify short segments by scanning the genome with a small window size. However as we mentioned earlier, these methods are limited in not being able to account for tumor purity and heterogeneity. It is worthwhile to incorporate alternative segmentation methods into the SomatiCA framework to identify SCNAs covering a much wider size distribution.

Thirdly, SomatiCA only supports subclonality characterization on one copy loss or gain because of the identifiability issue when subclonality and multiple-copy aberration coexists. This can be illustrated with the following toy example. Suppose there is a subclonal SCNA with 5 copies present in 30% of the cancer cells, then the expected somatic ratio (after adjusting for admixture rate) is 1.45. However, a SCNA with 4 copies present in 45% of the cancer cells and a SCNA with 3 copies present in 90% of the cancer cells all have exactly the same expected somatic ratio. Thus the testing only based on the somatic ratio can not make accurate inference about subclonality on multiple-copy aberrations. However, the copy number status for subclonal multiple-copy SCNAs may be estimated via a mixture component model on BAF. Subclonality characterization on multiple-copy SCNAs is another future direction to extend the SomatiCA framework.

Finally, in SomatiCA, tumor purity is estimated from copy number changes. In real applications, we suggest to compare the estimation from alternative approaches, such as PurityEst [26] and PurBayes [27], which estimate tumor purity based on the somatic single nucleotide aberrations. Moreover, SomatiCA assumes a single clonal cancer population and defines subclonality respect to the identified clonal cancer population. This assumption may be violated when there are multiple clonal cancer genomes within a sequencing profile. Here we note a method recently developed to address this problem, THetA [28], which supports deconvolution of the tumor genome mixture to a normal genome and any number of cancer genomes. However, the deconvolution results need to be interpreted with special caution to avoid overfitting.

## Materials and Methods

### Data preprocessing and GC bias correction

The BAM files of HCC1143 samples from TCGA benchmark 4 datasets were downloaded from https://cg hub.ucsc.edu/benchmark_download.html. In this study, we used 7 artificially mixed samples sequenced at 30× and 1 normal sample with the same coverage to compare against. Samtools [29] were used to call the genotypes from BAM files and filter out calls with quality score less than 10. Then we extracted RD information from corresponding VCF files and calculated LAF for each SNP. For the GLI-N1 sample, DNA extracted from a patient with diagnosed primary glioblastoma (GBM) was sequenced by Complete Genomics platform (unpublished data). RD was extracted directly from the MasterVarBeta file generated from Complete Genomics analysis pipeline and LAF was calculated by mirroring the BAF at 0.5.

SomatiCA corrects GC bias on RD using a linear regression model proposed by Diskin at el [30]. More specifically, SomatiCA selects SNPs whose RD ratio (RD/median of the library) as response variables based on the following criteria: 1) autosome SNPs only, 2) at least 1 Mb from each other to eliminate potential local dependence, 3) LAF greater than 0.4, read count ratio greater than 0.8 and less than 1.25 in both tumor and control samples to exclude the confounding of copy number on regression coefficients. The corrected RD ratio is the residual from the regression model.

### Signal smoothing to reduce the effects of outliers

Denote the observed LAF sequence as 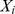, where 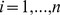 and 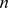 is total number of observed data points. The smoothing region for each 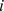 is given by 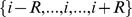, where the default 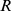 value is 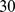. When the bounds as defined are out of range, the smoothing region is given by 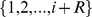 when 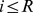 and the smoothing region is 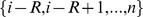 for 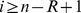. Let 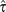 be the sample standard deviation of data in the smoothing region and let 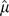 be the sample mean. If 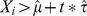 or 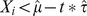, we replace 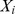 with median of that region. The default value for t is 2.

### CBS followed by LARS path based model selection

We used the CBS algorithm implemented in DNAcopy package [18] to segment the input LAF. We modified the original CBS segmentation procedure because the unsatisfied segmentation results on chromosomes without obvious change points. For an observed sequence 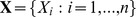, we add 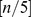 pseudo points 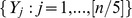 at the two ends of the sequence as a control for variation, where each 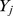 follows 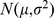 with 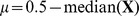 and 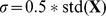. We first infer change points from the prolonged sequences then we removed pseudo segments and their associated change points.

After segmentation on the prolonged sequence, we apply a variable selection procedure to refine the inferred change points. More specifically, we model the input LAF as a piecewise constant regression model with 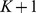 segments. LAF at position 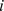 in the segment 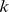 can be presented as a summation over mean shift levels before 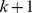-th segment and a noise component 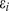 :
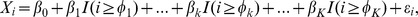
where 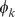 is the position for the 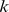-th change point, 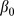 is the mean level for 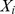 before the first change point, 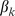 is the mean shift between the 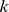-th and the 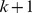-th segment and 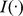 is the indicator function. Taking summation on both sides, we get the cumulative version of the above equation [20]:
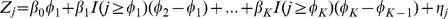
Where 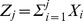. Given the input LAF sequence and 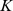 change points from initial segmentation 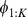, we use the step-wise regression implemented in LARS package [19] to estimate 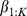. The LARS solution path provides an order for 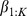 with each one's correlation with the residual increased. We select the first 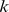 change points in the path as optimal change points via BIC, defined as 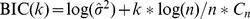, where 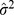 is residual variance, 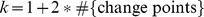 and 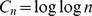 [20].

### Somatic ratio based on paired read depths

For each segment, we infer its associated somatic ratio based on RD of all paired SNPs (both heterozygous and homozygous sites) in that segment. From now on, we use the symbols 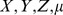 as new notations in this part. Let 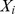 be the RD at the 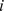-th position of that segment in the tumor sample, 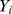 be the RD at this position of that segment in the normal sample. The distributions for each 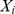 and 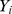 are believed to be Poisson distributed. For convenience of inference, we approximately model them by 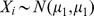 and 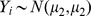. Let the ratio be 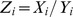, and then the Geary - Hinkley transformation
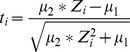
approximately follows a standard normal distribution. Let 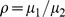 be the true somatic ratio in this segment, and we estimate 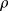 by the MLE
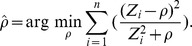
This problem can be solved by searching the optimum in 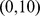. In the implementation, we exclude SNPs with 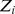 lower than the 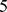-th percentile or larger than the 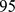-th percentile on each segment. SomatiCA also implements two alternative approaches to estimating the somatic ratio. One is accomplished by calculating the geometric mean of the RD ratios of all pairs in that segment. The other is accomplished by first calculating the MLE of 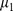 and 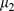, then 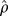 is estimated by 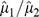. When the coverage from the two sequencing libraries is different, we provide an option for adjustment where RD from the tumor sample is adjusted so that the median RD values from the two libraries are identical.

### Admixture rate estimation by a Bayesian Finite Mixture model

SomatiCA models the somatic ratios of all segments using a Bayesian Finite Mixture model, with components centered at the discrete levels. Under a Bayesian framework, each segment is assigned with a discrete level based on the corresponding posterior probability. Segments with higher posterior probabilities are more likely to be clonal aberrations. Segments with ambiguous assignments, i.e, lower posterior probabilities, are classified as candidate subclonal events and excluded from admixture rate inference. The admixture rate is estimated by an optimal solution contributed by explanation of the copy number shift of clonal aberrations from integer levels.

Let us assume that the somatic copy levels (somatic ratio*2) consist of 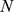 segments 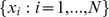. Each 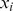 is assumed to have arisen from one of the 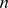 integer copy number states in the set 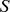. We define 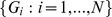 as indicators of copy number states. For each 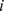, we model 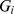 by
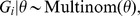
where 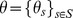 specify the expected fraction allocation to each copy-state. We use the conjugate prior of multinomial distribution, Dirichlet prior 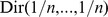 on 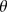, which means the allocation of the copy-states is mainly driven by the input data. Given 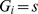, we model 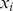 by
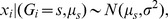
where 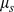 follows the prior
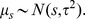
The number of components 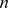 is estimated from the histogram of the somatic copy levels by the Akaike Information Criterion implemented in the REBMIX algorithm [31]. To avoid overfitting, we require the centers of the components have a distance of at least 0.2 (corresponding to 80% normal contamination). The minimum number of components is set to 3 (a scenario with no change, one copy loss and one copy gain).

Under the above model, the posterior probability of 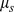 is given by:
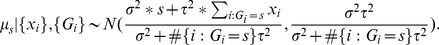
The posterior distribution of 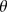 given observations follows 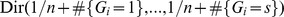. We set hyperparameters 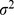 and 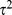 equal to 0.01, i.e., we allow the copy level of clonal events shifting from integer levels at about 0.1. For example, the segment with somatic copy level of 1.85 has high probability been assigned with integer copy number of 2. However, the segment with that of 1.5 could be assigned with integer copy number of 1 or 2 since its shift from integer levels are much greater than 0.1. This ambiguous assignment could be reflected as a low posterior probability with integer copy number of 1or 2. It will be classified as potential subclonal events and excluded from the estimation of admixture rate.

We have implemented a Metropolis-Hasting algorithm to infer the allocation of copy-states. We use 10,000 iterations with the first 2000 as burn-in to calculate the posterior probabilities. The 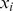
's with lower posterior probabilities in the copy-states allocation are denoted as candidate subclonal segments. Denote the set of these candidate subclonal segments by 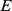. Then we use segments not in 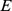 to estimate the admixture rate by
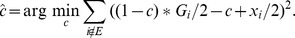



### Hypothesis testing based subclonality characterization

Based on GC corrected read count ratio (R), SomatiCA calculates allelic copy number for each segment in the normal sample. Define 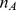 and 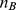 to be the copy numbers for two alleles in that segment,
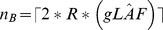


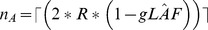
where 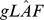 is the median germline LAF on that segment. Given 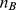 and 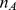 in a normal sample, SomatiCA tests whether copy number change in the corresponding tumor sample can result in a change of exactly one copy of one allele.

If the somatic ratio 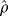 (corrected by admixture rate) in the corresponding tumor sample is greater than 1, SomatiCA tests for one copy gain with theoretical clonal copy number ratio 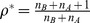; otherwise it tests for one copy loss with 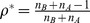. With the null hypothesis that clonal copy number ratio follows a normal distribution 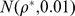, p-value is calculated for each segment as the probability of obtaining a copy number ratio at least as extreme as the one that was actually observed 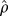. Segments with p-value less than 0.05 are classified as subclonal. The percentage of tumor cells with subclonal change can be further calculated by
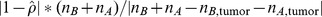
where integer allelic copy numbers in tumor sample 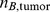 and 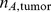 are estimated as
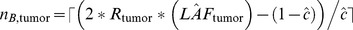


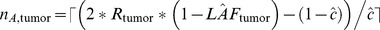
and 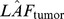 is the median LAF on that segment in the tumor sample.

## Supporting Information

Figure S1 **An example showing the effect of denoising step in SomatiCA.**
(PNG)

Figure S2 **The estimation from SomatiCA helped to increase the accuracy of the inferred copy number inference for SCNAs compared to setting admixture rate at pre-specified (and incorrect) levels.**
(PDF)

Figure S3 **Comparison of segmentation methods on Chromosome 7 of a GBM sample.**
(PDF)

Figure S4 **Comparison of segmentation methods on Chromosome 10 of a GBM sample.**
(PDF)

Text S1 **This document consists of scripts generating simulated data and running ABSOLUTE etc.**
(ZIP)

Text S2 **This document provides a detailed tour of the usage of SomatiCA package.**
(PDF)
