# Supplementary figures and images for: SomatiCA: Identifying, Characterizing and Quantifying Somatic Copy Number Aberrations from Cancer Genome Sequencing Data
Source: PLoS One. 2013 Nov 12;8(11):e78143. doi: 10.1371/journal.pone.0078143 (PMC3827077; doi:10.1371/journal.pone.0078143)

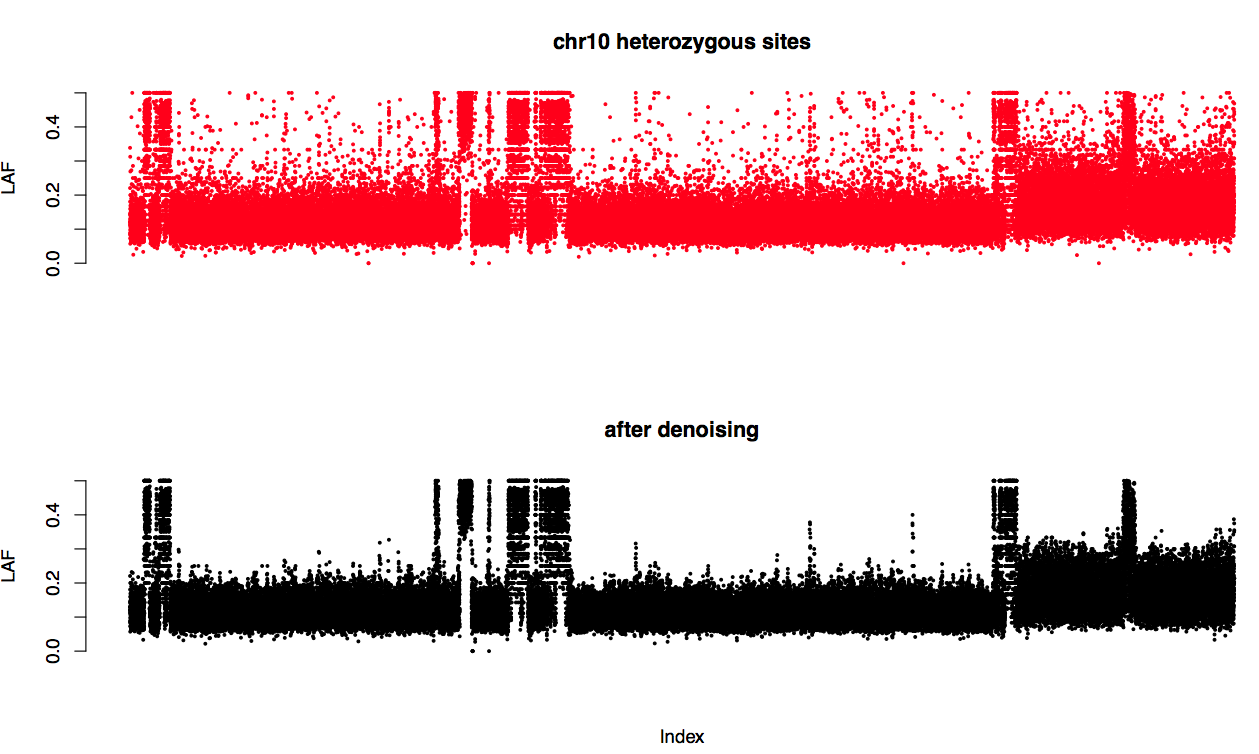

Supplement: Figure S1 — An example showing the effect of denoising step in SomatiCA. (PNG) [file pone.0078143.s001.png]

Accuracy

Coverage = 20X

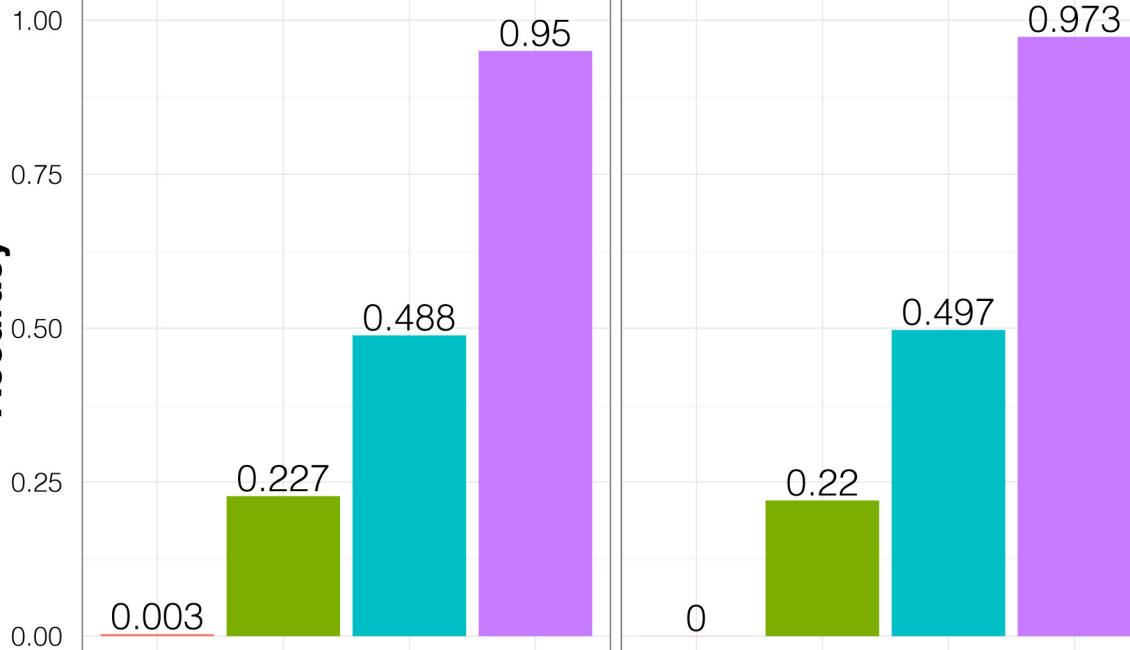

Coverage = 40X

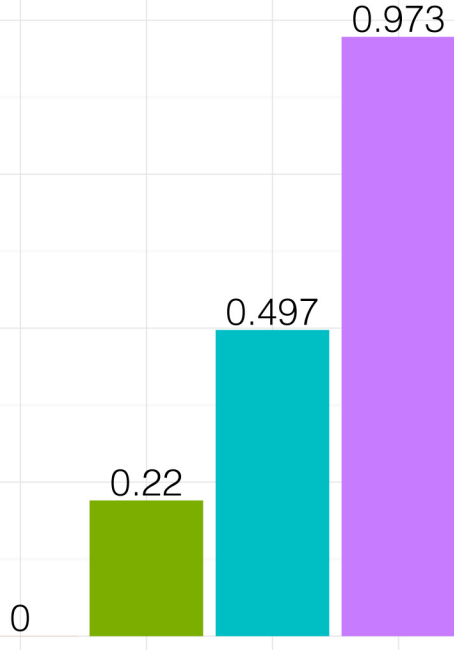

Coverage = 60X

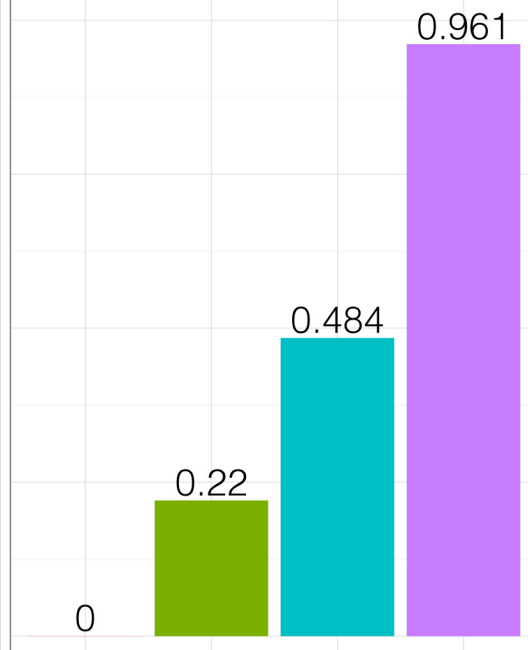

Adjustment

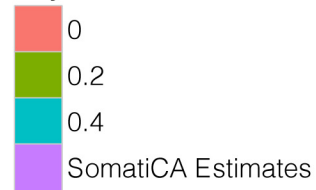

Supplement: Figure S2 — The estimation from SomatiCA helped to increase the accuracy of the inferred copy number inference for SCNAs compared to setting admixture rate at pre-specified (and incorrect) levels. (PDF) [file pone.0078143.s002.pdf]

**CBS**

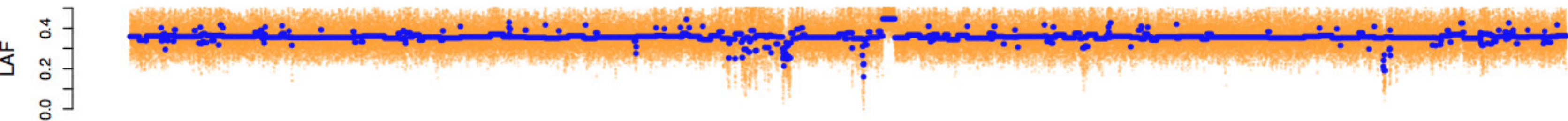

**cumSeg**

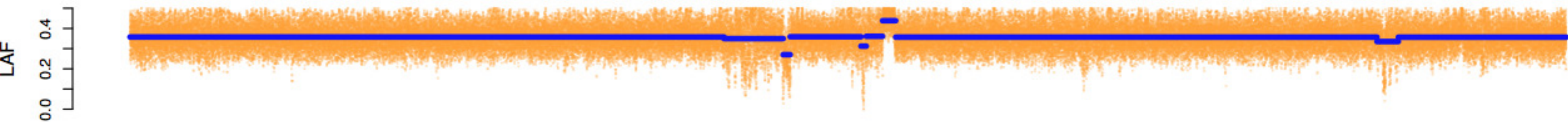

**SomatiCA**

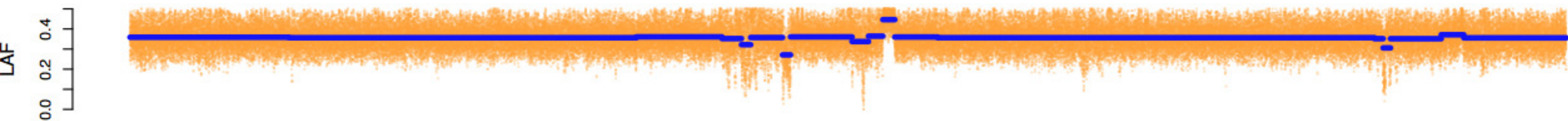

Supplement: Figure S3 — Comparison of segmentation methods on Chromosome 7 of a GBM sample. (PDF) [file pone.0078143.s003.pdf]

**CBS**

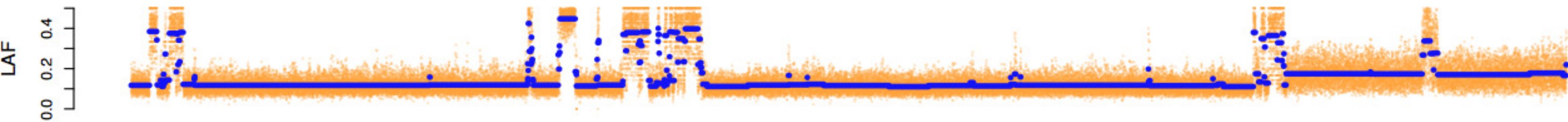

**cumSeg**

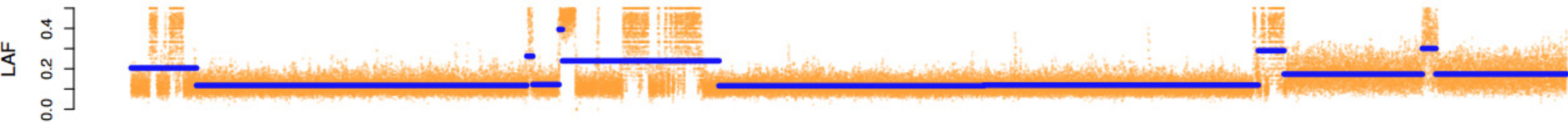

**SomatiCA**

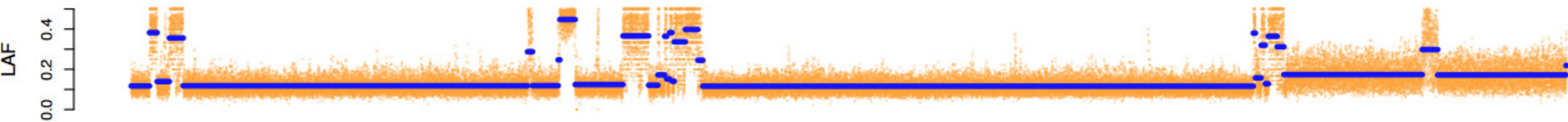

Supplement: Figure S4 — Comparison of segmentation methods on Chromosome 10 of a GBM sample. (PDF) [file pone.0078143.s004.pdf]
