# Supplementary material for: SomatiCA: Identifying, Characterizing and Quantifying Somatic Copy Number Aberrations from Cancer Genome Sequencing Data
Source: PLoS One. 2013 Nov 12;8(11):e78143. doi: 10.1371/journal.pone.0078143 (PMC3827077; doi:10.1371/journal.pone.0078143)
Supplement: Text S2 — This document provides a detailed tour of the usage of SomatiCA package. (PDF) [file pone.0078143.s006.pdf]

SomatiCA: identifying, characterizing and quantifying somatic copy number aberrations from cancer genome sequencing

User's Guide

Mengjie Chen, Hongyu Zhao

Yale University

June 3, 2013

1 Introduction

1.1 Scope

This guide provides a tour of the Bioconductor package `SomatiCA`, a R package that is capable of identifying, characterizing and quantifying somatic CNAs from cancer whole genome sequencing. It is especially designed for somatic copy number analyses taking into account: (i) an unknown fraction of normal cells (admixture rate) that are nearly always intermixed with cancer cells; and (ii) the heterogeneity of cancer cell population owing to ongoing subclonal evolution. The package implements a pipeline for characterizing somatic copy number aberrations based on different statistical methods: segmentation on Lesser Allele Frequency (LAF), Maximum Likelihood Estimation of somatic ratio (read depth ratio of tumor/ normal), admixture rate estimation by a Bayesian finite mixture model and subclonality characterization based on hypothesis testing. It is especially suitable for studies designed to understand tumor evolution. It currently works for cancer sample with control.

This guide begins with overview of each module in the pipeline, and then gives an example of fully worked case study.

1.2 Quick start

A typical *SomatiCA* analysis using the implemented pipeline look like the following. We assume that there are two sequencing libraries with tumor and matched control, and the LAF and read depths are stored in a tab-delimited text file, with position and zygosity annotated (see dataset glio in SomatiCAData).

```
> library(SomatiCA)
> library(SomatiCAData)
```

```
> x <- read.table("tumor_sample_with_control.txt", header=T, sep="\t")
> input <- SomatiCAFormat(x, missing = T, verbose = T)
```

```
> data(GCcontent)
> segments <- SomatiCApipe(input, ncores = 1, collapse.k = 0,
+                           method = "mle", mcmc = 10000, burnin = 5000,
+                           p = 0.001, GC=GCcontent)
```

Users could also apply each step in the pipeline separately as demonstrated below.

2 Overview of capabilities

2.1 Input

SomatiCAdata stored a sample data `glio` from whole genome sequencing, with 3458745 SNPs on 24 chromosomes (including chrX, chrM).

```
> data(glio)
> head(glio)

  chromosome   pos zygosity totalReadCount        LAF
1       chr1 38232      hom              3 0.33333333
2       chr1 38907      hom             11 0.36363636
3       chr1 41981      hom              7 0.14285714
4       chr1 46670  het-ref             38 0.05263158
5       chr1 47108  het-ref             21 0.00000000
6       chr1 47292  het-ref             51 0.21568627
  totalReadCountN germlineLAF
1               2   0.5000000
2              16   0.4375000
3              10   0.5000000
4              28   0.1071429
5              22   0.1363636
6              47   0.2127660
```

We first limit our analysis on a portion of chromosome 10 for illustration purposes. `SomatiCAFormat()` will format the above data frame into input for SomatiCA pipeline, which is a GRange instance.

```
> glio_sub <- glio[glio[, 1]%in%c("chr10"), ]
> colnames(glio_sub) <- c("seqnames", "start", "zygosity", "tCount",
+                         "LAF", "tCountN", "germLAF")
> input <- SomatiCAFormat(glio_sub, missing = T, verbose = T)
```

If the data frame is big, removing missing values may take a while. If missing values have been removed in previous pre-processing steps, use `missing = F` instead to reduce computing time.

2.2 Segmentation

Given input in the format of a GRanges object, `larsCBSsegment()` segments each chromosome with LAF of heterozygous sites on that chromosomes. `larsCBSsegment()` extracts hetergozygous sites by 'grep' any site with 'het' in the column of zygosity. For Complete Genomic data, sites with zygosity of 'het-ref' and 'het-alt' will be extracted. If genotype calling results are obtained from other platforms, a transformation is needed. For example, the '0/1/2' coding from VCF file will be needed to transform to 'het'/'hom' coding. Users can name 'zygosity' in their own way but keep in mind that only names containing 'het' will be used as heterozygous sites for segmentations in SomatiCA, such as 'het', 'het-ref', 'heter', 'heter1' etc.

`larsCBSsegment()` firstly calls a function `denoise()` to smooth the outliers. Then it segment each chromosome with CBS followed by a model selection procedure. The default model selection criteria is Bayesian Information Criterion (BIC). Users can set `rss=TRUE` to apply BIC plus a minimum cut-off for change in residue sum of squares (RSS) between neighboring change points. `collapse.k` is an option to average LAF on each k SNPs.

```
> seg <- larsCBSsegment(input, collapse.k = 0, ncores = 1, verbose = T,
+                        rss=T, S=0.5)
```

Output of `larsCBSsegment()` includes two part: segmentation results and heterozygous sites used for segmentation (denoised).

```
> seg

$segment
GRanges with 31 ranges and 2 elementMetadata values:
       seqnames                 ranges strand   |    medLAF
          <Rle>              <IRanges>  <Rle>   | <numeric>
   [1]    chr10   [        0,  1647315]      *  |    0.1136
   [2]    chr10   [ 1647315,  1980728]      *  |       0.4
   [3]    chr10   [ 1980728,  2497863]      *  |    0.1212
   [4]    chr10   [ 2497863,  3339647]      *  |       0.4
   [5]    chr10   [ 3339647, 37807847]      *  |    0.1132
   [6]    chr10   [37807847, 42753044]      *  |    0.2727
   [7]    chr10   [42753044, 46153563]      *  |    0.1125
   [8]    chr10   [46153563, 46791048]      *  |       0.2
   [9]    chr10   [46791048, 47148128]      *  |    0.4555
   ...      ...                    ...    ... ...       ...
  [23]    chr10 [115430816, 115879467]      *  |      0.14
  [24]    chr10 [115879467, 116113366]      *  |    0.3636
  [25]    chr10 [116113366, 116310102]      *  |    0.1151
  [26]    chr10 [116310102, 116775885]      *  |       0.4
  [27]    chr10 [116775885, 117577995]      *  |    0.3333
  [28]    chr10 [117577995, 127540392]      *  |    0.1687
  [29]    chr10 [127540392, 127616678]      *  |    0.3065
  [30]    chr10 [127616678, 135421344]      *  |    0.1667
```

```
  [31]    chr10 [135421344, 135506780]      *   |    0.1972
        medgLAF
      <numeric>
   [1]    0.4495
   [2]    0.4444
   [3]    0.4493
   [4]      0.44
   [5]    0.4531
   [6]    0.3158
   [7]    0.4516
   [8]    0.3263
   [9]    0.3824
   ...       ...
  [23]    0.4508
  [24]    0.4432
  [25]    0.4505
  [26]    0.4352
  [27]    0.4251
  [28]    0.4545
  [29]     0.298
  [30]    0.4507
  [31]    0.4545
  ---
  seqlengths:
   chr10
      NA

$hetsites
GRanges with 71887 ranges and 5 elementMetadata values:
          seqnames                 ranges strand   |
             <Rle>              <IRanges>  <Rle>   |
      [1]    chr10       [ 81245,  81245]      *   |
      [2]    chr10       [110655, 110655]      *   |
      [3]    chr10       [118119, 118119]      *   |
      [4]    chr10       [124381, 124381]      *   |
      [5]    chr10       [132768, 132768]      *   |
      [6]    chr10       [133341, 133341]      *   |
      [7]    chr10       [133803, 133803]      *   |
      [8]    chr10       [135656, 135656]      *   |
      [9]    chr10       [141909, 141909]      *   |
      ...      ...                    ...    ... ...
  [71879]    chr10 [135434551, 135434551]      *   |
  [71880]    chr10 [135437185, 135437185]      *   |
  [71881]    chr10 [135499455, 135499455]      *   |
  [71882]    chr10 [135499729, 135499729]      *   |
```

```
[71883]    chr10 [135499771, 135499771]      *  |
[71884]    chr10 [135499781, 135499781]      *  |
[71885]    chr10 [135506185, 135506185]      *  |
[71886]    chr10 [135506687, 135506687]      *  |
[71887]    chr10 [135506780, 135506780]      *  |
           zygosity    tCount       LAF   tCountN
        <character> <integer> <numeric> <integer>
    [1]     het-ref        62 0.3387097        75
    [2]     het-ref        68 0.2205882        94
    [3]     het-ref        57 0.1228070        55
    [4]     het-ref        83 0.1807229       103
    [5]     het-ref        71 0.1126761        81
    [6]     het-ref        35 0.1428571        50
    [7]     het-ref        25 0.1200000        27
    [8]     het-ref        31 0.1290323        74
    [9]     het-ref        69 0.1594203       100
    ...         ...       ...       ...       ...
[71879]     het-ref        60 0.2000000       116
[71880]     het-ref        20 0.3500000        33
[71881]     het-ref        24 0.3750000        22
[71882]     het-ref        19 0.4210526        19
[71883]     het-ref        13 0.3076923         9
[71884]     het-ref         9 0.4444444        11
[71885]     het-ref        64 0.4687500        97
[71886]     het-ref        48 0.4375000        68
[71887]     het-ref        62 0.3548387       104
          germLAF
        <numeric>
    [1] 0.3600000
    [2] 0.5000000
    [3] 0.4727273
    [4] 0.4951456
    [5] 0.4444444
    [6] 0.4800000
    [7] 0.4444444
    [8] 0.4729730
    [9] 0.4200000
    ...       ...
[71879] 0.4310345
[71880] 0.3030303
[71881] 0.2727273
[71882] 0.4210526
[71883] 0.3333333
[71884] 0.4545455
[71885] 0.4020619
```

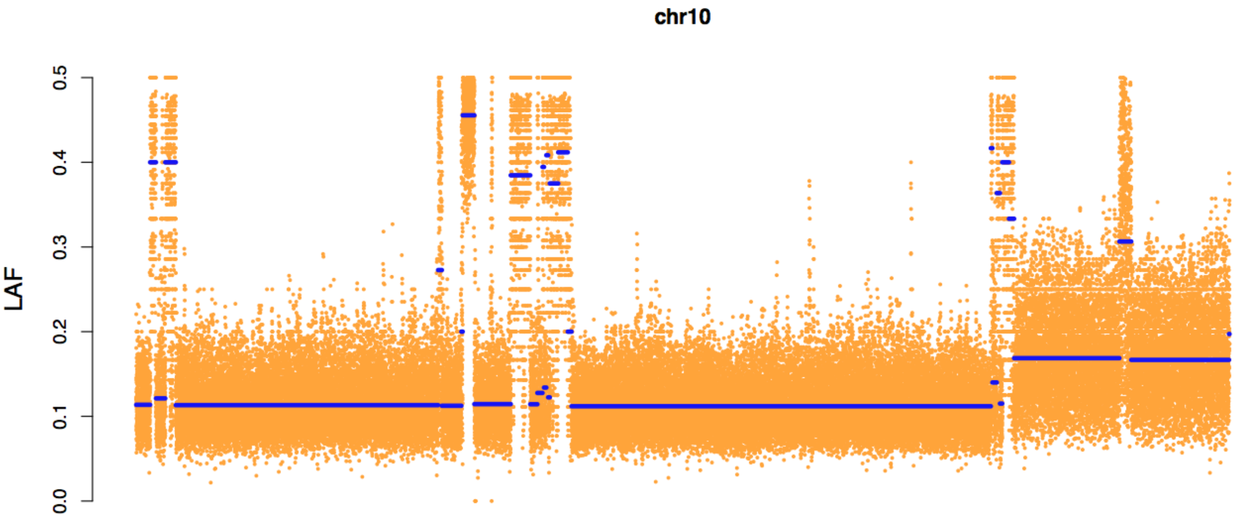

Figure 1: Segmentation on chr10 based on LAF (denoised data).

```
[71886] 0.4558824
[71887] 0.1923077
---
seqlengths:
 chr10
    NA
```

The segmentation result can be examined by `plotSegment()`. If multiple chromosomes are segmented, use `k` to plot segmentation on each chromosome respectively by their orders in the input. Use use `smooth`=T to visualize denoised data. This segmentation is not final. It will be refined based on somatic ratio.

```
> plotSegment(seg$segment, input, k = 1, smooth = T)
```

`larsCBSsegment()` supports multi-thread parallel computing through package ***doParallel*** and ***foreach***. The number of cores used in computing could be specified by argument `ncores`. Before parallel computing, we recommend to check the usable cores and register for multiple cores as the following example.

```
> require(doMC)
> registerDoMC(4)
> k <- getDoParWorkers()
> k
> seg <- larsCBSsegment(input, collapse.k = 0, ncores = k, verbose = T,
+                         rss=T, S=0.5)
```

2.3 Estimation of somatic ratio

Somatic ratio is defined as the ratio of read depths between a tumor and its paired normal sample for a given segment. SomatiCA implements different methods to estimate somatic ratio. For the "mle" method somatic ratio is estimated by a maximum likelihood approach. For the "mean" method, somatic ratio is estimated by the ratio between mean of tumor sample and normal sample. For the "geometric", somatic ratio is estimated by geometric mean of somatic ratios of all sites in a given segment. To estimate somatic ratio, both segmentation and input with read depths are required.

```
> segmentwithRatio <- somaticRatio(seg$segment, input, method = "mle")
> segmentwithRatio

GRanges with 31 ranges and 3 elementMetadata values:
       seqnames                 ranges strand   |    medLAF
          <Rle>              <IRanges>  <Rle>   | <numeric>
   [1]    chr10   [       0,  1647315]      *   |    0.0641
   [2]    chr10   [ 1647315,  1980728]      *   |    0.2857
   [3]    chr10   [ 1980728,  2497863]      *   |    0.0857
   [4]    chr10   [ 2497863,  3339647]      *   |     0.125
   [5]    chr10   [ 3339647, 37807847]      *   |    0.0556
   [6]    chr10   [37807847, 42753044]      *   |    0.0274
   [7]    chr10   [42753044, 46153563]      *   |    0.0397
   [8]    chr10   [46153563, 46791048]      *   |    0.0822
   [9]    chr10   [46791048, 47148128]      *   |    0.4272
   ...      ...                    ...    ... ...       ...
  [23]    chr10 [115430816, 115879467]      *   |    0.0667
  [24]    chr10 [115879467, 116113366]      *   |    0.3333
  [25]    chr10 [116113366, 116310102]      *   |    0.0781
  [26]    chr10 [116310102, 116775885]      *   |     0.375
  [27]    chr10 [116775885, 117577995]      *   |      0.02
  [28]    chr10 [117577995, 127540392]      *   |     0.093
  [29]    chr10 [127540392, 127616678]      *   |    0.2203
  [30]    chr10 [127616678, 135421344]      *   |    0.1096
  [31]    chr10 [135421344, 135506780]      *   |    0.1574
         medgLAF     ratio
       <numeric> <numeric>
   [1]    0.3793     0.895
   [2]    0.3837     0.181
   [3]       0.4     0.772
   [4]    0.0312     0.183
   [5]    0.3286     0.797
   [6]     0.017     0.757
   [7]    0.0398     0.857
   [8]    0.0801     0.744
   [9]    0.2696     0.904
```

```
  ...       ...        ...
 [23]    0.0445     0.586
 [24]    0.4375     0.197
 [25]    0.4067     0.866
 [26]    0.4333     0.155
 [27]     0.014     0.253
 [28]    0.3623     0.529
 [29]     0.257     0.791
 [30]    0.3857     0.568
 [31]     0.266     0.608
 ---
 seqlengths:
  chr10
     NA
```

2.4 Refine segments

Two adjacent segments are merged if the difference in the somatic ratios is less than "threshold2", which is tunable in the implementation with its default value being 0.05, equivalent to 5% change in somatic copy-number without normal contamination. The MLEs of the somatic ratio for the refined segments are recalculated. This refinement procedure is applied repeatedly until no adjacent segments have somatic ratio difference less than T. "threshold1" is the threshold used to merge the segments based on median LAF.

```
> refined <- refineSegment(segmentwithRatio, input, method = "mle",
+                          adjust = TRUE, threshold1 = 0 , threshold2 = 0.05)
> refined
```

```
GRanges with 25 ranges and 3 elementMetadata values:
       seqnames                 ranges strand   |    medLAF
          <Rle>              <IRanges>  <Rle>   | <numeric>
   [1]    chr10   [       0,  1647315]      *   |    0.0641
   [2]    chr10   [ 1647315,  1980728]      *   |    0.2857
   [3]    chr10   [ 1980728,  2497863]      *   |    0.0857
   [4]    chr10   [ 2497863,  3339647]      *   |     0.125
   [5]    chr10   [ 3339647, 42753044]      *   |    0.0556
   [6]    chr10   [42753044, 46153563]      *   |    0.0397
   [7]    chr10   [46153563, 46791048]      *   |    0.0822
   [8]    chr10   [46791048, 54101613]      *   |    0.0741
   [9]    chr10   [54101613, 55558759]      *   |      0.25
  ...       ...                    ...    ... ...       ...
  [17]    chr10 [115360452, 115430816]      *   |    0.4118
  [18]    chr10 [115430816, 115879467]      *   |    0.0667
  [19]    chr10 [115879467, 116113366]      *   |    0.3333
  [20]    chr10 [116113366, 116310102]      *   |    0.0781
```

```
[21]    chr10 [116310102, 116775885]      *   |     0.375
[22]    chr10 [116775885, 117577995]      *   |      0.02
[23]    chr10 [117577995, 127540392]      *   |     0.093
[24]    chr10 [127540392, 127616678]      *   |    0.2203
[25]    chr10 [127616678, 135506780]      *   |    0.1111
       medgLAF     ratio
     <numeric> <numeric>
 [1]    0.3793     1.218
 [2]    0.3837     0.246
 [3]       0.4     1.044
 [4]    0.0312     0.248
 [5]    0.3125     1.082
 [6]    0.0398     1.165
 [7]    0.0801     0.983
 [8]    0.2697     1.186
 [9]    0.3256     0.223
 ...       ...       ...
[17]    0.4529     0.248
[18]    0.0445     0.791
[19]    0.4375      0.26
[20]    0.4067      1.18
[21]    0.4333     0.211
[22]     0.014     0.325
[23]    0.3623      0.72
[24]     0.257     1.079
[25]    0.3846     0.772
---
seqlengths:
 chr10
    NA
```

2.5 Estimation of admixture rate

The estimation of the admixture rate is accomplished by fitting the somatic copy number (somatic ratio*2) of all segments with a Bayesian finite mixture model, with components centered at the discrete levels. Each segment was assigned with a discrete level based on corresponding posterior probability. Segments with ambiguous assignments will be classified as candidate subclonal events and excluded from admixture rate inference. The admixture rate will be estimated by an optimal solution contributed by explanation of tumor copy number with all remaining segments as integer level. ***SomatiCA*** implements a Markov Chain Monte Carlo with Metropolis Hasting algorithm to estimate posterior probabilities of the Bayesian finite mixture model. Option `mcmc` set the number of MCMC iteration, `burnin` set the number of MCMC iteration for burnin and `p` set the cutoff of posterior probability for ambiguous integer copy number assignments.

```
> data(segwithratio)
> segmentwithRatio <-  GRanges(seqnames=segwithratio$chromosome,
+               ranges=IRanges(start=segwithratio$start,
+                              end=segwithratio$end),
+               medLAF=segwithratio$medLAF,
+               medgLAF=segwithratio$medgermlineLAF,
+               ratio=segwithratio$ratio)
> x <- admixtureRate(segmentwithRatio, mcmc = 5000, burnin = 1000, p = 0.01)
> admix <- x$admix
```

copynumberCorrected could take a segmentation profile and an admixture rate to calculate the integer somatic copy number in tumor sample then characterize somatic events based on corrected somatic ratio. Each segment will be annotated with ”=”, ”Loss”, ”Gain”, ”LOH”, ”neutral LOH” or ”double deletion”. Note that somatic copy number here is calculated from the ratio with the assumption that the control sample is diploid. This result will likely be modified in next step with the calculation of ploidy of each segment in the control sample.

```
> y <- copynumberCorrected(segmentwithRatio, admix)
> y
```

```
GRanges with 137 ranges and 5 elementMetadata values:
        seqnames                 ranges strand   |
           <Rle>              <IRanges>  <Rle>   |
    [1]     chr1 [        0,   4364422]      *   |
    [2]     chr1 [  4364422,   4767753]      *   |
    [3]     chr1 [  4767753,   7301947]      *   |
    [4]     chr1 [  7301947,  16886985]      *   |
    [5]     chr1 [ 16886985,  18141145]      *   |
    [6]     chr1 [ 18141145,  45869770]      *   |
    [7]     chr1 [ 45869770,  54958545]      *   |
    [8]     chr1 [ 54958545, 120391484]      *   |
    [9]     chr1 [120391484, 145381187]      *   |
    ...      ...                    ...    ... ...
  [129]    chr17  [72633298,  81194665]      *   |
  [130]    chr18  [       0,  78016752]      *   |
  [131]    chr19  [       0,  59102379]      *   |
  [132]    chr20  [       0,  62964366]      *   |
  [133]    chr21  [       0,  48097460]      *   |
  [134]    chr22  [       0,  17040586]      *   |
  [135]    chr22  [17040586,  51195304]      *   |
  [136]     chrX  [       0,  82705277]      *   |
  [137]     chrX  [82705277, 155257115]      *   |
           medLAF   medgLAF     ratio  somaCN       event
        <numeric> <numeric> <numeric> <array> <character>
    [1] 0.4431818 0.4444444     1.117       2           =
    [2] 0.3513645 0.4526316     0.885       2           =
```

```
    [3]  0.1714286 0.4482759     0.627       1         LOH
    [4]  0.4464286 0.4489796     0.999       2           =
    [5]  0.3444976 0.3529412     1.022       2           =
    [6]  0.4492140 0.4516129     1.009       2           =
    [7]  0.4409722 0.4512195     1.015       2           =
    [8]  0.4491275 0.4533333     0.962       2           =
    [9]  0.3778653 0.3697479     1.013       2           =
    ...        ...       ...       ...     ...         ...
  [129] 0.44680851 0.4468085     1.141       2           =
  [130] 0.45028549 0.4528302     0.984       2           =
  [131] 0.37837838 0.4461538     1.153       2           =
  [132] 0.45000000 0.4523810     1.025       2           =
  [133] 0.44683421 0.4482759     0.941       2           =
  [134] 0.21126761 0.2550263     0.756       1        Loss
  [135] 0.09756098 0.4495413     1.242       3        Gain
  [136] 0.44444444 0.4473684     0.887       2           =
  [137] 0.44721485 0.4500000     0.889       2           =
  ---
  seqlengths:
    chr1 chr10 chr11 chr12 chr13 ...  chr7  chr8  chr9  chrX
      NA    NA    NA    NA    NA ...    NA    NA    NA    NA

>
```

2.6 Subclonality characterization

SomatiCA estimates subclonality for each somatic copy number abberation. To do this, it first calculates allelic copy number nB and nA (segment level allelic copy number is estimated by median in that segment) in a control sample based on GC corrected read counts. SomatiCA tests whether copy number change in corresponding tumor sample can result in a change of exactly one copy of one allele. If the somatic ratio (corrected by admixture rate) in the corresponding tumor sample is greater than 1, SomatiCA tests for one copy gain, otherwise it tests for one copy loss. With null hypothesis that clonal copy number ratio follows a normal distribution, p-value is calculated for each segment as the probability of obtaining a copy number ratio at least as extreme as the one that was actually observed. Segments with p-value less than 0.05 are classified as subclonal.

```
> data(glio)
> colnames(glio) <- c("seqnames", "start", "zygosity", "tCount", "LAF", "tCountN", "ge
> input <- SomatiCAFormat(glio, missing = T, verbose = T)
> data(GCcontent)
> segmentGCcorrected <- segmentGCbiasRemoval(y, input, GCcontent)
> segmentClonality <- subclonality(segmentGCcorrected, admix)
> segmentClonality
```

```
GRanges with 137 ranges and 9 elementMetadata values:
        seqnames                 ranges strand   |
           <Rle>              <IRanges>  <Rle>   |
    [1]     chr1 [        0,   4364422]      *   |
    [2]     chr1 [  4364422,   4767753]      *   |
    [3]     chr1 [  4767753,   7301947]      *   |
    [4]     chr1 [  7301947,  16886985]      *   |
    [5]     chr1 [ 16886985,  18141145]      *   |
    [6]     chr1 [ 18141145,  45869770]      *   |
    [7]     chr1 [ 45869770,  54958545]      *   |
    [8]     chr1 [ 54958545, 120391484]      *   |
    [9]     chr1 [120391484, 145381187]      *   |
    ...      ...                    ...    ... ...
  [129]    chr17  [72633298,  81194665]      *   |
  [130]    chr18  [       0,  78016752]      *   |
  [131]    chr19  [       0,  59102379]      *   |
  [132]    chr20  [       0,  62964366]      *   |
  [133]    chr21  [       0,  48097460]      *   |
  [134]    chr22  [       0,  17040586]      *   |
  [135]    chr22  [17040586,  51195304]      *   |
  [136]     chrX  [       0,  82705277]      *   |
  [137]     chrX  [82705277, 155257115]      *   |
            medLAF   medgLAF     ratio  somaCN       event
         <numeric> <numeric> <numeric> <array> <character>
    [1]  0.4431818 0.4444444 1.1833856       2           =
    [2]  0.3513645 0.4526316 0.8197492       2           =
    [3]  0.1714286 0.4482759 0.4153605       1         LOH
    [4]  0.4464286 0.4489796 0.9984326       2           =
    [5]  0.3444976 0.3529412 1.0344828       2           =
    [6]  0.4492140 0.4516129 1.0141066       2           =
    [7]  0.4409722 0.4512195 1.0235110       2           =
    [8]  0.4491275 0.4533333 0.9404389       2           =
    [9]  0.3778653 0.3697479 1.0203762       2           =
    ...        ...       ...       ...     ...         ...
  [129] 0.44680851 0.4468085 1.2210031       2           =
  [130] 0.45028549 0.4528302 0.9749216       2           =
  [131] 0.37837838 0.4461538 1.2398119       2           =
  [132] 0.45000000 0.4523810 1.0391850       2           =
  [133] 0.44683421 0.4482759 0.9075235       2           =
  [134] 0.21126761 0.2550263 0.6175549       1        Loss
  [135] 0.09756098 0.4495413 1.3793103       3        Gain
  [136] 0.44444444 0.4473684 0.8228840       2           =
  [137] 0.44721485 0.4500000 0.8260188       2           =
           clonality    germCN subclonalCN subpercent
         <character> <numeric>   <numeric>  <numeric>
```

```
   [1]              =         2          2        0.0
   [2] subclonal_loss         2          1        0.3
   [3]         clonal         2          1        1.0
   [4]              =         2          2        0.0
   [5]              =         2          2        0.0
   [6]              =         2          2        0.0
   [7]              =         2          2        0.0
   [8]              =         2          2        0.0
   [9]              =         4          4        0.0
   ...            ...       ...        ...        ...
 [129]              =         2          2        0.0
 [130]              =         2          2        0.0
 [131] subclonal_gain         2          3        0.4
 [132]              =         2          2        0.0
 [133]              =         2          2        0.0
 [134]         clonal         2          1        1.0
 [135]         clonal         2          3        1.0
 [136]              =      <NA>       <NA>        0.0
 [137]              =      <NA>       <NA>        0.0
 ---
 seqlengths:
   chr1 chr10 chr11 chr12 chr13 ...  chr7  chr8  chr9  chrX
     NA    NA    NA    NA    NA ...    NA    NA    NA    NA
```

Users can further merge neighboring segments with same somatic copy number and events together.

```
> merged <- MergeSegment(segmentClonality)
> merged
```

```
GRanges with 93 ranges and 6 elementMetadata values:
       seqnames                 ranges strand   |
          <Rle>              <IRanges>  <Rle>   |
   [1]     chr1 [        0,   4364422]      *   |
   [2]     chr1 [  4364422,   4767753]      *   |
   [3]     chr1 [  4767753,   7301947]      *   |
   [4]     chr1 [  7301947, 120391484]      *   |
   [5]     chr1 [120391484, 145381187]      *   |
   [6]     chr1 [145381187, 249239305]      *   |
   [7]     chr2 [        0, 133037397]      *   |
   [8]     chr2 [133037397, 133100540]      *   |
   [9]     chr2 [133100540, 230315858]      *   |
   ...      ...                    ...    ... ...
  [85]    chr17  [27292044,  29539325]      *   |
  [86]    chr17  [29539325,  81194665]      *   |
  [87]    chr18  [       0,  78016752]      *   |
```

```
[88]    chr19  [        0,  59102379]      *   |
[89]    chr20  [        0,  62964366]      *   |
[90]    chr21  [        0,  48097460]      *   |
[91]    chr22  [        0,  17040586]      *   |
[92]    chr22  [17040586,  51195304]      *   |
[93]     chrX  [        0, 155257115]      *   |
           somaCN       event      clonality    germCN
      <character> <character>    <character> <integer>
 [1]            2           =              =         2
 [2]            2           = subclonal_loss         2
 [3]            1         LOH         clonal         2
 [4]            2           =              =         2
 [5]            2           =              =         4
 [6]            2           =              =         2
 [7]            2           =              =         2
 [8]            2           =              =         4
 [9]            2           =              =         2
 ...          ...         ...            ...       ...
[85]            1         LOH         clonal         2
[86]            2           =              =         2
[87]            2           =              =         2
[88]            2           = subclonal_gain         2
[89]            2           =              =         2
[90]            2           =              =         2
[91]            1        Loss         clonal         2
[92]            3        Gain         clonal         2
[93]            2           =              =      <NA>
      subclonalCN subpercent
        <integer>  <numeric>
 [1]            2        0.0
 [2]            1        0.3
 [3]            1        1.0
 [4]            2        0.0
 [5]            4        0.0
 [6]            2        0.0
 [7]            2        0.0
 [8]            4        0.0
 [9]            2        0.0
 ...          ...        ...
[85]            1        1.0
[86]            2        0.0
[87]            2        0.0
[88]            3        0.4
[89]            2        0.0
[90]            2        0.0
```

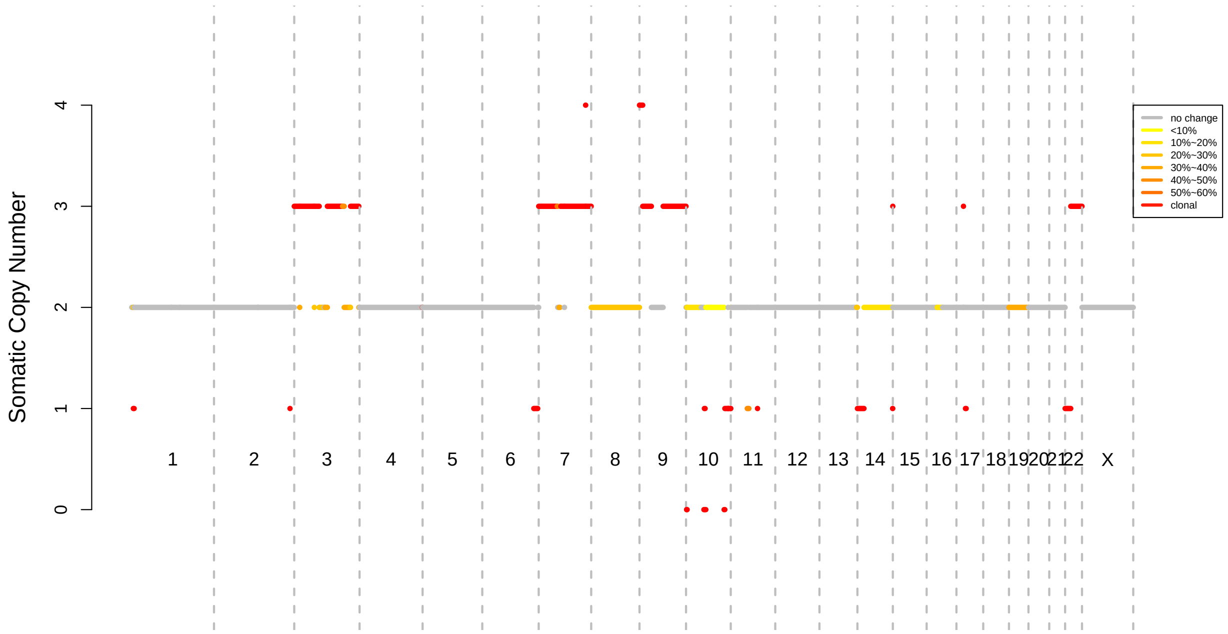

Figure 2: An example of subclonality.

```
[91]            1        1.0
[92]            3        1.0
[93]         <NA>        0.0
---
seqlengths:
  chr1 chr10 chr11 chr12 chr13 ...  chr7  chr8  chr9  chrX
    NA    NA    NA    NA    NA ...    NA    NA    NA    NA
```

The subclonality and somatic copy number for a sample can be visualized by `plotSubclonality`.

```
> plotSubclonality(merged)
```

Then you can output all the clonal events.

```
> merged[elementMetadata(merged)[, "clonality"]=="clonal", ]

GRanges with 34 ranges and 6 elementMetadata values:
       seqnames                 ranges strand   |
          <Rle>              <IRanges>  <Rle>   |
   [1]     chr1 [  4767753,   7301947]      *   |
   [2]     chr2 [230315858, 230370805]      *   |
   [3]     chr3 [        0,  16396272]      *   |
   [4]     chr3 [ 16675587,  60744470]      *   |
   [5]     chr3 [ 60934640,  75695612]      *   |
   [6]     chr3 [100385706, 146381696]      *   |
```

```
  [7]     chr3 [195317386, 195734615]      *   |
  [8]     chr4 [190471562, 191038383]      *   |
  [9]     chr6 [156062524, 168058043]      *   |
  ...      ...                    ...    ... ...
 [26]    chr10 [135422545, 135506780]      *   |
 [27]    chr11 [ 81029748,  81234533]      *   |
 [28]    chr14 [        0,  19878708]      *   |
 [29]    chr14 [106531090, 107149006]      *   |
 [30]    chr14 [107149006, 107287496]      *   |
 [31]    chr17 [ 21201550,  21352174]      *   |
 [32]    chr17 [ 27292044,  29539325]      *   |
 [33]    chr22 [        0,  17040586]      *   |
 [34]    chr22 [ 17040586,  51195304]      *   |
           somaCN       event   clonality    germCN
      <character> <character> <character> <integer>
  [1]           1         LOH      clonal         2
  [2]           1         LOH      clonal         2
  [3]           3        Gain      clonal         2
  [4]           3        Gain      clonal         2
  [5]           3        Gain      clonal         2
  [6]           3        Gain      clonal         2
  [7]           3        Gain      clonal         2
  [8]           2        Gain      clonal         4
  [9]           1         LOH      clonal         2
  ...         ...         ...         ...       ...
 [26]           1        Loss      clonal         2
 [27]           1         LOH      clonal         2
 [28]           1        Loss      clonal         2
 [29]           1        Loss      clonal         2
 [30]           3        Gain      clonal         2
 [31]           3        Gain      clonal         4
 [32]           1         LOH      clonal         2
 [33]           1        Loss      clonal         2
 [34]           3        Gain      clonal         2
      subclonalCN subpercent
        <integer>  <numeric>
  [1]           1          1
  [2]           1          1
  [3]           3          1
  [4]           3          1
  [5]           3          1
  [6]           3          1
  [7]           3          1
  [8]           5          1
  [9]           1          1
```

```
  ...          ...        ...
  [26]           1          1
  [27]           1          1
  [28]           1          1
  [29]           1          1
  [30]           3          1
  [31]           6          1
  [32]           1          1
  [33]           1          1
  [34]           3          1
  ---
  seqlengths:
    chr1 chr10 chr11 chr12 chr13 ...  chr7  chr8  chr9  chrX
      NA    NA    NA    NA    NA ...    NA    NA    NA    NA
```

Or output the subclonal events with high proportion of aberration.

```
> merged[elementMetadata(merged)[, "subpercent"]>0.3, ]

GRanges with 46 ranges and 6 elementMetadata values:
       seqnames                 ranges strand   |
          <Rle>              <IRanges>  <Rle>   |
   [1]     chr1 [  4767753,   7301947]      *   |
   [2]     chr2 [230315858, 230370805]      *   |
   [3]     chr3 [        0,  16396272]      *   |
   [4]     chr3 [ 16396272,  16675587]      *   |
   [5]     chr3 [ 16675587,  60744470]      *   |
   [6]     chr3 [ 60934640,  75695612]      *   |
   [7]     chr3 [ 93522442, 100385706]      *   |
   [8]     chr3 [100385706, 146381696]      *   |
   [9]     chr3 [146381696, 151423319]      *   |
   ...      ...                    ...    ... ...
  [38]    chr11 [ 81029748,  81234533]      *   |
  [39]    chr14 [        0,  19878708]      *   |
  [40]    chr14 [106531090, 107149006]      *   |
  [41]    chr14 [107149006, 107287496]      *   |
  [42]    chr17 [ 21201550,  21352174]      *   |
  [43]    chr17 [ 27292044,  29539325]      *   |
  [44]    chr19 [        0,  59102379]      *   |
  [45]    chr22 [        0,  17040586]      *   |
  [46]    chr22 [ 17040586,  51195304]      *   |
            somaCN       event      clonality    germCN
       <character> <character>    <character> <integer>
   [1]           1         LOH         clonal         2
   [2]           1         LOH         clonal         2
   [3]           3        Gain         clonal         2
```

```
 [4]           2       Loss subclonal_loss         2
 [5]           3       Gain         clonal         2
 [6]           3       Gain         clonal         2
 [7]           2          = subclonal_gain         2
 [8]           3       Gain         clonal         2
 [9]           3       Gain subclonal_gain         2
 ...         ...        ...            ...       ...
[38]           1        LOH         clonal         2
[39]           1       Loss         clonal         2
[40]           1       Loss         clonal         2
[41]           3       Gain         clonal         2
[42]           3       Gain         clonal         4
[43]           1        LOH         clonal         2
[44]           2          = subclonal_gain         2
[45]           1       Loss         clonal         2
[46]           3       Gain         clonal         2
     subclonalCN subpercent
       <integer>  <numeric>
 [1]           1        1.0
 [2]           1        1.0
 [3]           3        1.0
 [4]           1        0.4
 [5]           3        1.0
 [6]           3        1.0
 [7]           3        0.4
 [8]           3        1.0
 [9]           3        0.5
 ...         ...        ...
[38]           1        1.0
[39]           1        1.0
[40]           1        1.0
[41]           3        1.0
[42]           6        1.0
[43]           1        1.0
[44]           3        0.4
[45]           1        1.0
[46]           3        1.0
---
seqlengths:
  chr1 chr10 chr11 chr12 chr13 ...  chr7  chr8  chr9  chrX
    NA    NA    NA    NA    NA ...    NA    NA    NA    NA
```

3 SomatiCA pipeline

Already get familiar with how `SomatiCA` works? If so, call `SomatiCApipe` directly to run all the steps described above automatically. Multithread computing is still supported through `ncores`. We recommend to use `verbose=T` to print working messages and track the working progress.

```
> data(GCcontent)
> x <- SomatiCApipe(input, ncores = 1, collapse.k = 0, method = "mle",
+                   mcmc = 50000, burnin = 10000, p = 0.001, GC = GCcontent)
> merged <- MergeSegment(x$segment)
```

4 Others

4.1 GC content

In case the precalculated GC content will be out of date or users may want to use smaller window size, we provide a function first downloading the .fa.gz of a given chromosome from UCSC genome browser and then calculating the GC content for a given window size (10,000 bp in the following example).

```
> chr <- paste("chr", c(1:22, "X"), sep="")
> url <- "http://hgdownload.soe.ucsc.edu/goldenPath/hg19/chromosomes/"
> GC <- foreach(i=chr, .combine=rbind)%dopar%{
+     return(GCcount(i, 10000, url))
+ }
```
